# Synthesis, Computational, and Anticancer In Vitro Investigations of Aminobenzylnaphthols Derived from 2-Naphtol, Benzaldehydes, and α-Aminoacids via the Betti Reaction

**DOI:** 10.3390/molecules28207230

**Published:** 2023-10-23

**Authors:** Mateusz Kciuk, Martyna Malinowska, Adrianna Gielecińska, Rajamanikandan Sundaraj, Somdutt Mujwar, Anna Zawisza, Renata Kontek

**Affiliations:** 1 University of Lodz, Faculty of Biology and Environmental Protection, Department of Molecular Biotechnology and Genetics, Banacha St. 12/16, 90-237 Lodz, Poland; 2 University of Lodz, Doctoral School of Exact and Natural Sciences, Banacha St. 12/16, 90-237 Lodz, Poland; 3 University of Lodz, Department of Organic and Applied Chemistry, Tamka 12, 91-403 Lodz, Poland; 4 Centre for Drug Discovery, Department of Biochemistry, Karpagam Academy of Higher Education, Coimbatore 641021, Tamil Nadu, India; 5 Chitkara College of Pharmacy, Chitkara University, Rajpura 140401, Punjab, India

**Keywords:** anticancer, apoptosis, Betti bases, cytotoxicity, molecular docking, molecular dynamics

## Abstract

Multicomponent reactions have emerged as an important approach for the synthesis of diverse and complicated chemical compounds. They have various advantages over two-component reactions, including the convenience of one-pot procedures and the ability to modify the structure of agents. Here, we employed in vitro and in silico studies to explore the anticancer potential of novel aminobenzylnaphthols derived from the Betti reaction (**MMZ** compounds). MTT assay was used to explore the cytotoxic activity of the compounds in pancreatic (BxPC-3 cells) and colorectal (HT-29) cancer cell lines or normal human lung fibroblasts (WI-38 cells). Proapoptotic properties of two derivatives **MMZ-45AA** and **MMZ-140C** were explored using AO/EB and annexin V-FITC/PI staining. In silico studies including ADMET profiling, molecular target prediction, docking, and dynamics were employed. The compounds exhibited cytotoxic properties and showed proapoptotic properties in respective IC_50_ concentrations. As indicated by in silico investigations, anticancer activity of **MMZs** can be attributed to the inhibition of ADORA1, CDK2, and TRIM24. Furthermore, compounds exhibited favorable ADMET properties. **MMZs** constitute an interesting scaffold for the potential development of new anticancer agents.

## 1. Introduction

Cancer is a significant worldwide health challenge. Different types of cancer, and even individual tumors, can have distinct characteristics that influence their susceptibility to therapeutical agents. New medications with increased effectiveness can be developed by selectively tailoring the drug against molecular targets involved in tumor growth, metastasis, and the development of resistance mechanisms. Many currently available anticancer medications have considerable side effects that might impair patients’ quality of life. The goal of new drug development is to reduce adverse effects while preserving or improving therapeutic efficacy. Search for new anticancer medications is critical for boosting efficacy, minimizing side effects, overcoming resistance, and promoting the development of effective combination therapies. These activities are critical in the continuous fight against cancer, intending to save lives and improve the quality of life for cancer patients [1,2,3,4].

Computer-assisted drug design (CADD) is a multidisciplinary area that uses computational methods and technologies to aid in the discovery and development of novel medications. It combines many computational tools, molecular modeling, and simulation methodologies to speed drug development and optimize the features of possible therapeutic candidates. CADD plays an important role in numerous facets of anticancer drug design. CADD enables the screening of vast chemical libraries of molecules that have the potential to interact with a specific target of interest. Virtual screening approaches, such as molecular docking and molecular dynamics simulations, facilitate the prediction of small molecule binding affinity and mode of association with target proteins. This allows researchers to prioritize and pick the most promising compounds for future experimental evaluation, saving time and money on experimental screening. CADD approaches may aid in the optimization of features such as potency, selectivity, and pharmacokinetic parameters once prospective drug candidates have been identified. Furthermore, molecular modeling and simulation techniques can help to improve binding affinity, enhance drug-likeness, and predict the absorption, distribution, metabolism, excretion, and toxicity (ADMET) features of substances. This iterative method also sheds light on the molecular mechanisms underlying drug–target interactions and drives the development of more effective and safe medicines [5,6,7,8,9].

Multicomponent reactions (MCRs) have emerged as a critical method for the synthesis of varied and complex chemical compounds. They offer several inherent advantages over two-component reactions, including the ease of one-pot techniques and the possibility of structural modification. Synthetic competency is derived from several tandem bond formation processes in MCRs, which conserve time, energy, and raw material. The Betti reaction, a modified version of the Mannich reaction, has become important in synthetic chemistry due to the production of C-C bonds under light experimental conditions [10].

In the Betti condensation reaction, aminobenzylnaphthols are synthesized from 2-naphthol, aryl aldehydes, and amines [11]. It was discovered in the early 20th century by the Italian chemist Mario Betti and was forgotten for decades until 1998 when it was brought back to life [12]. Since then, the Betti reaction has returned to the interest of organic chemists around the world and is now experiencing its second youth. Aminobenzylnaphthols formed in this transformation, the so-called Betti bases, are a class of molecules found in many natural and synthetic compounds with a wide range of interesting activities and applications [13]. Betti base derivatives were tested as antitumor agents [14], sodium- and chloride-dependent neutral and basic amino acid transporter B(0+) (SLC6A14) blockers [13], antiyeast agents inhibiting Candida albicans growth [15], antitumor and antioxidants [16], and multidrug resistance (MDR) reversal agents [17].

**Figure 1:**
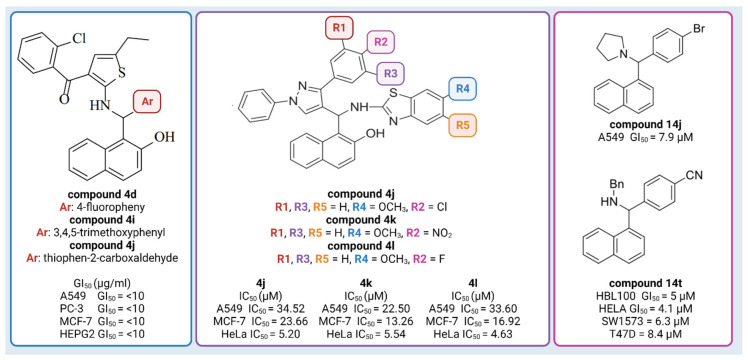
Examples of Betti compounds with profound antiproliferative activity against various cell lines. Created with Biorender.com, accessed on 6 March 2023.

So far, naphthol derivatives have been investigated for bioactivity or employed as building blocks in drug discovery. For example, Yellapurkar et al. [16] synthesized thiophene containing aminobenzylnaphthols. Three of them (**compound 4d**, **compound 4i**, and **compound 4j**) exhibited profound anticancer activity (GI_50_ 10 μg/mL) against four cancer cell lines (A549 (lung), PC-3 (prostate), MCF-7 (breast) and HEPG2 (liver)), which was equivalent to the standard agent doxorubicin (Figure 1) [16]. Nagaraju et al. [18] developed a new class of pyrazole-linked benzothiazole–naphthol derivatives. Three compounds (**compound 4j, 4k, 4l**) showed significant cytotoxicity against human cervical cancer cells (HeLa), with IC_50_ values ranging from 4.63 to 5.54 μM (Figure 1). Flow cytometry examination demonstrated that these derivatives caused cell cycle arrest in the G2/M phase, and spectroscopic experiments such as UV–visible, fluorescence, and circular dichroism studies suggested that these compounds had a high affinity for binding DNA. Furthermore, these compounds efficiently suppressed the activity of topoisomerase I. Most notably, compounds **102**, **103**, and **104** inhibited topoisomerase I at 100 μM concentrations, similar to known topoisomerase inhibitor-camptothecin. Furthermore, compounds demonstrated promising cytotoxic and antiproliferative activity against the human cervical cancer cell line (HeLa), with IC_50_ values ranging from 4.63 to 5.54 μM [18]. Puerta et al. [13] synthesized a library of 23 Betti bases and tested their cytotoxic activities against human breast cancer cell lines (A549, HBL-100, T-47D), human cervical cancer cell line (HeLa), alveolar cell carcinoma (SW1573), and human colon cancer cell line (WiDr). **Compound 14j** had the highest antiproliferative activity against the human breast cancer cell line A549, with GI_50_ values of 7.9 μM, while the reference drug (cisplatin) had a GI_50_ value of 4.9 µM. **Compound 14t** was found to have significant antiproliferative action, with GI_50_ values in the micromolar range: HBL100 (5 μM), HeLa (4.1 μM), SW1573 (6.3 μM), and T47D (8.4 μM) (Figure 1) [13]. Furthermore, docking experiments demonstrated that Betti bases disrupt the SLC6A14 solute transporter, resulting in amino acid deprivation and suppressing the proliferation of cells by acting as tryptophan mimetics [13].

Recently, there has been renewed interest in the use of amino acid derivatives as prodrugs. The functionalization of a medicine with an amino acid residue leads to certain advantages, such as improved drug transport to the target area and reduced toxicity. Boroxazolidones, for example, were shown to have limited anticancer characteristics; nevertheless, l-valine derivatives had substantial cytotoxic effects. l-valine-modified dimethyl-curcumin demonstrated significantly more antiproliferative activity than the original drugs, differentiating itself as a strong anticancer agent [19,20,21]. The functionalization of the drug with an amino acid moiety results in positive effects, such as improved delivery of the drug to the target tissue and reduction in its toxicity. In addition, such compounds show stronger cytotoxic and anticancer properties [20,21] compared to derivatives without an amino acid group. However, to the best of our knowledge, there is only one example of testing aminoacid-functionalized Betti base as an antitumor agent [19].

Here, we report a synthesis of new aminobenzylnaphthols (**MMZ** compounds) obtained by the Betti reaction using methyl derivatives of (*R*)-valine, (*S*)-phenylalanine, (*S*)-proline, (*S*)-glutamic acid, (*S*)-aspartic acid, and (*S*)-leucine. The resulting derivatives, in the form of a mixture of enantiomers (*S*,*S*) and (*R*,*R*) or (*R*,*S*) and (*S*,*R*), were subjected to cytotoxic and proapoptotic properties evaluation. These studies were complemented with extensive prediction of compound properties, including absorption, distribution, metabolism, excretion and toxicity features (ADMET), chemical reactivity, kinetic structural stability, and electronic properties, followed by in silico target prediction, molecular docking, and investigation of the stability of the best target-compound complexes.

## 2. Results

### 2.1. Chemistry

Synthesis of MMZ Compounds

**Figure 2:**
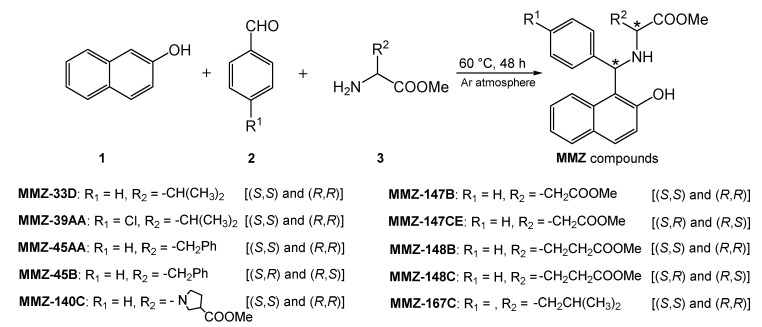
Scheme of the Betti reaction with aminoacid derivatives.

We have recently subjected chiral d- and l-amino acid methyl esters to the Betti reaction to obtain a library of new amino acid derivatives of Betti bases, the **MMZ** compounds (Figure 2), which appear to be of interest over a wide range of bioactivities. Note that we present first examples of Betti reaction with methyl derivatives of (*R*)-waline, (*S*)-phenyl alanine, (*S*)-proline, (*S*)-glutamic acid, (*S*)-aspartic acid, and (*S*)-leucine.

It was a solvent-free, green synthetic process where 2-naphthol (**1**), aryl aldehydes **2,** and aminoacid methyl esters **3** reacted at 60 °C to yield corresponding aminobenzylnaphtols, according to a protocol reported in the literature [22].

**Figure 3:**
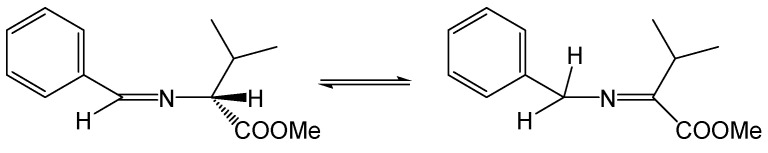
Aza-allyl tautomerism in an aldimine derived from (*R*)-valine methyl ester.

Reaction without solvent and at elevated temperature has disadvantages. It turns out that the stereogenic center of the amino acid, which theoretically should not be involved in the reaction, is not fixed under these conditions, and a mixture of (*S*,*S*) and (*R*,*R*) enantiomers is formed as the main product [22]. This is due to the aza-allyl tautomerization to which the imine is formed from an aldehyde and an amino acid ester (Figure 3).

The amino acid derivatives can undergo racemization in the presence of not only elevated temperature, but also of aldehydes [23]. In our work, we occasionally isolated small amounts of the (*R*,*S*) and (*S*,*R*)-stereoisomers [24]. The (*S*,*S*) and (*R*,*R*)-stereoisomers, because of their crystal stability, present in larger quantities and they are the first eluting in chromatographic separations. Right afterward, in some cases, we were able to isolate a mixture of (*R*,*S*) and (*S*,*R*)-stereoisomers. However, since the 1H-NMR spectra of the stereoisomers (*R*,*S*)/(*S*,*R*) and (*S*,*S*)/(*R*,*R*) have characteristic, nonoverlapping patterns, they can be used to distinguish them. According to the literature, high-field signals are characteristic of (*S,S*)/(*R*,*R*), and low-field signals are characteristic of (*R*,*S*)/(*S*,*R*) stereoisomers, which was also reflected in our case [24,25]. Small values of optical rotation and quite high values of melting points indicate that we are aware that this is a mixture of (*S*,*S*)/(*R*,*R*) and (*R*,*S*)/(*S*,*R*)-enantiomers. This was not considered as an obstacle and we decided to continue biological research on mixtures. Due to the poor solubility of compounds **MMZ-148B** and **MMZ-148C**, they have not been further investigated, but we present them as compounds previously unknown in the literature. The NMR spectra are included in Supplementary Figures S1–S10 (SM1.pdf file).

### 2.2. Biological Studies

#### 2.2.1. MTT Assay

**Figure 4:**
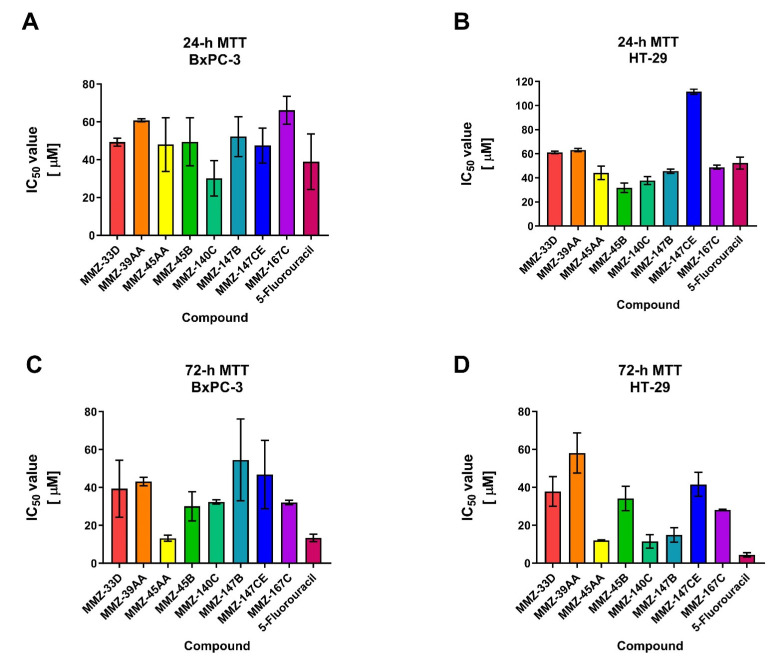
Results of 24 h (**A**,**B**) and 72 h (**C**,**D**) MTT assay: mean IC_50_ ± SD values obtained for **MMZ-33D**, **MMZ-49AA**, **MMZ-45AA**, **MMZ-45B**, **MMZ-140C**, **MMZ-147B**, **MMZ-147CE,** and **MMZ-167C** compounds, and 5-Fluorouracil in BxPC-3 and HT-29 cells.

The 24 and 72 h MTT assay was used to determine the cytotoxic properties of **MMZ** compounds used in the concentration range 5–400 µM. The investigation revealed that all of the **MMZ** compounds tested in the current study possessed cytotoxic activity toward BxPC-3 and HT-29 cells. The activity of Betti bases and their derivatives have not been previously tested in BxPC-3 and HT-29 cells, therefore these cell lines were selected for the initial evaluation of their anticancer potential. 5-Fluorouracil was used as a positive control in the experiment, given its application in the treatment of both pancreatic and colorectal cancers [26,27,28]. The results are shown in Figure 4 and Supplementary Table S1 (SM1.pdf file).

Following 24 h treatment of BxPC-3 cells with **MMZ** compounds, the mean IC_50_ values varied between 30.15 ± 9.39 µM (**MMZ-140C**) and 66.19 ± 7.36 (**MMZ-167C**). The cytotoxic activity of compound **MMZ-140C** was comparable to this observed for 5-Fluorouracil (IC_50_ = 38.99 ± 14.67) (Figure 4A). In HT-29 cells, treatment with **MMZ** derivatives resulted in the cytotoxic effects in the range 31.78 ± 3.93 µM (**MMZ-45B**) to 111.5 ± 2.12 µM (**MMZ-147CE**). The cytotoxic effects of **MMZ-45B** and **MMZ-140C** (37.76 ± 3.2 µM) were comparable and exceeded this observed for 5-Fluorouracil (IC_50_ = 52.26 ± 4.9) (Figure 4B).

Following 72 h, the mean IC_50_ values varied between 13.26 μM (**MMZ-45AA**) and 54.55 μM (**MMZ-147B**) in BxPC-3 cells. **MMZ-45B**, **MMZ-140C**, and **MMZ-167C** exhibited similar cytotoxicity with mean IC_50_ values between 30.13 and 32.42 μM. The cytotoxic activity of **MMZ-45AA** was comparable to this observed for 5-Fluorouracil (IC_50_ = 13.43 ± 1.9 µM) (Figure 4C). In the HT-29 cell line, IC_50_ values varied between 11.55 μM (**MMZ-140C**) and 58.11 μM (**MMZ-39AA**). The cytotoxic activity of **MMZ** compounds did not exceeded this observed for 5-Fluorouracil (IC_50_ = 4.38 ± 1.1 µM) (Figure 4D).

**Figure 5:**
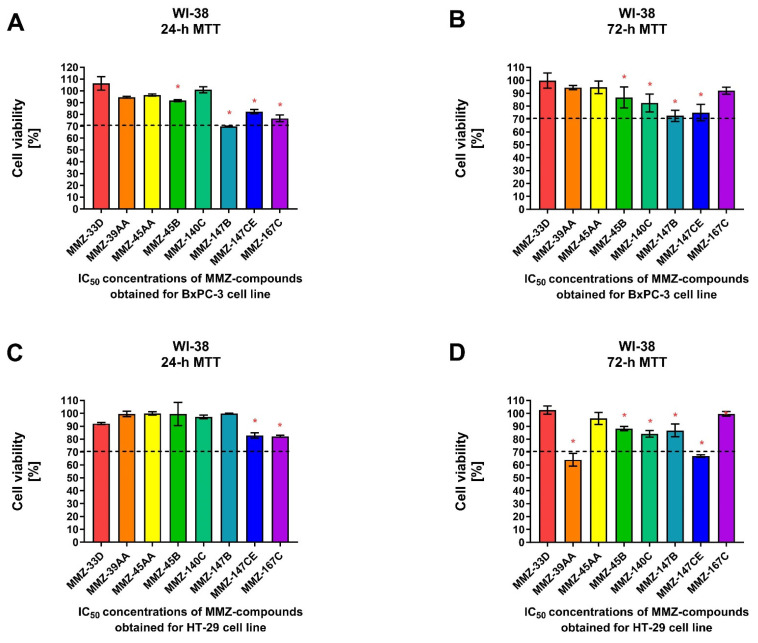
Cell viability of normal human lung fibroblast cells (WI-38) after 24 and 72 h treatment with **MMZ** compounds used in IC_50_ concentrations obtained in the MTT assay for BxPC-3 (**A**,**B**) and HT-29 (**C**,**D**) cell lines following respective 24 or 72 h incubation times. Data are presented as the percentage of cell viability (% cell viability ± SD). The differences between the experimental samples and control group (100% viability/DMSO-treated) were evaluated by the ANOVA test followed by Dunnett’s test. Asterisk (*) indicates significant difference compared to the control group.

Additionally, we used the human fibroblast cell line WI-38 to determine the effects of **MMZ** compounds on normal cell viability following 24 and 72 h treatment with IC_50_ concentrations of the compounds previously obtained for BxPC-3 and HT-29 cells following respective incubation times (Figure 5).

For 24 h incubation time, a statistically significant (*p* < 0.05) decrease in WI-38 cell viability was observed following treatment with **MMZ-45B** (91.73 ± 0.87%; *p* = 0.0388), **MMZ-147B** (69.94 ± 0.21%; *p* < 0.0001), **MMZ-147CE** (82.45 ± 1.8%; *p* = 0.0003), and **MMZ167C** (76.77 ± 1.8%; *p* < 0.0001) compounds used in IC_50_ concentrations estimated for BxPC-3 cells. Only **MMZ-147B** reduced cell viability below 70% cell viability threshold (Figure 5A). In contrast, **MMZ-45B** (86.71 ± 8.14%; *p* = 0.03), **MMZ-140C** (82.32 ± 6.96%; *p* = 0.0037), **MMZ-147B** (72.45 ± 4.37%; *p* < 0.0001), and **MMZ-147CE** (74,93 ± 6.4%; *p* < 0.0001) compounds induced a statistically significant (*p* < 0.05) decrease in cell viability following 72 h incubation time. However, the incubation of WI-38 cells with the tested compounds did not resulted in decrease in cell viability below 70% (Figure 5B).

Following 24 h incubation of WI-38 cells with compounds in IC_50_ concentrations obtained for HT-29 cells, only **MMZ-140C** (82.90 ± 2.06%; *p* = 0.0031) and **MMZ-167C** (82.19 ± 0.69%; *p* = 0.0023) caused a statistically significant loss in WI-38 cell viability (Figure 5C), while a more pronounced response was observed following 72 h incubation, where **MMZ-39AA** (64.03 ± 4.9%; *p* < 0.0001), **MMZ-45B** (88.2 ± 1.6%; *p* = 0.0021), **MMZ-140C** (84.15 ± 2.56%; *p* < 0.0001), **MMZ-147B** (86.8 ± 5%; *p* = 0.0006), and **MMZ-147CE** (66.9 ± 1.08%; *p* < 0.0001) led to a decrease in normal human cell viability. Nevertheless, only **MMZ-147CE** suppressed WI-38 cell viability below 70% (Figure 5D).

Given the observed cytotoxic activity of **MMZ** compounds **MMZ-45AA** and **MMZ-140C** derivatives and their effects on normal cell viability, these compounds were selected for further investigation.

#### 2.2.2. Dual Acridine Orange/Ethidium Bromide (AO/EB) Fluorescent Staining

**Figure 6:**
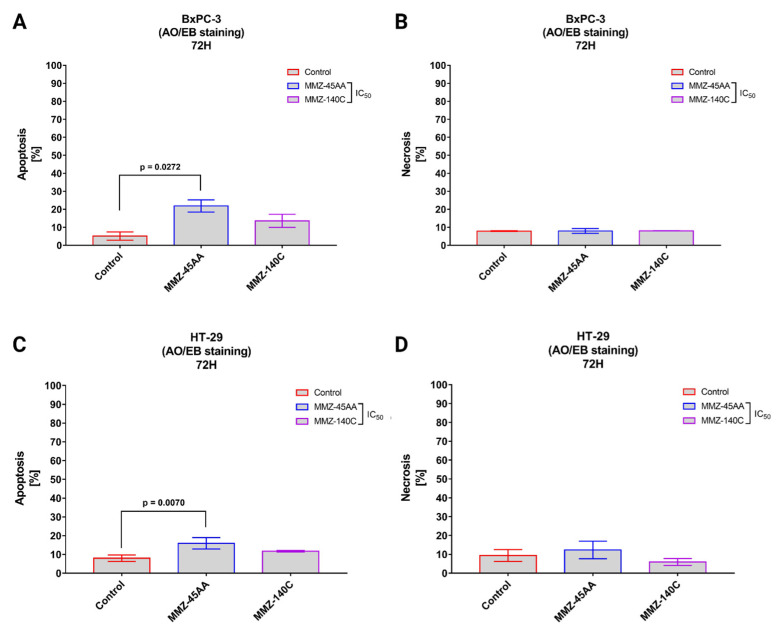
Apoptosis and necrosis induction in BxPC-3 cells (**A**,**B**) and HT-29 cells (**C**,**D**) treated with IC_50_ concentrations of **MMZ45-AA** and **MMZ-140C** compounds measured with AO/EB double staining following 72 h incubation time. Data are presented as mean percentage of apoptotic cells (early and late apoptotic) or necrotic cells ± SD values. The differences between the experimental samples and (untreated) control were evaluated by the ANOVA test followed by Tukey’s test (*p* < 0.05); N = 200.

Dual Acridine Orange/Ethidium Bromide (AO/EB) combines the distinct uptake of fluorescent DNA-binding dyes AO and EB with the morphologic feature of chromatin condensation in the stained nucleus to differentiate between live, apoptotic, and necrotic cells [29]. **MMZ-45AA** and **MMZ-140C** were chosen for further studies and analyzed for apoptosis and necrosis induction in BxPC-3 (Figure 6A,B) and HT-29 cells (Figure 6C,D).

In BxPC-3 cells (Figure 6A), a statistically significant (*p* = 0.0272) increase in apoptotic cell fraction (% of apoptotic cell = 21.9 ± 3.4%) was observed in cells treated with IC_50_ concentration of **MMZ-45AA** compared to a negative control group (% of apoptotic cell = 5.15 ± 2.33%). No statistically significant changes (*p* = 0.15) in apoptotic cell fraction were observed for cells treated with IC_50_ concentration of **MMZ-140C**. Similarly, no statistically significant (*p* = 0.97) changes in the mean number of necrotic cells were observed among experimental groups (Figure 6B).

In HT-29 cells (Figure 6C), a statistically significant (*p* = 0.007) increase in apoptotic cell fraction was observed following treatment of cells with IC_50_ concentration of **MMZ-45AA** (% of apoptotic cells = 15.93 ± 3%) compared with control cells (% of apoptotic cell = 8 ± 1.73%). No statistically significant increase (*p* = 0.14) in the number of apoptotic cells was observed following treatment with the **MMZ-140C** compound used in IC_50_ concentration (% of apoptotic cells = 11.73 ± 0.38%). No statistically significant increase (*p* < 0.05) in the necrotic fraction was observed following the treatment of HT-29 cells with the two **MMZ** compounds used in IC_50_ concentrations (Figure 6D). The final solvent concentration of DMSO was < 0.5% *v/v* in all experimental samples.

#### 2.2.3. Annexin V-FITC and Propidium Iodide Flow Cytometry Analysis

**Figure 7:**
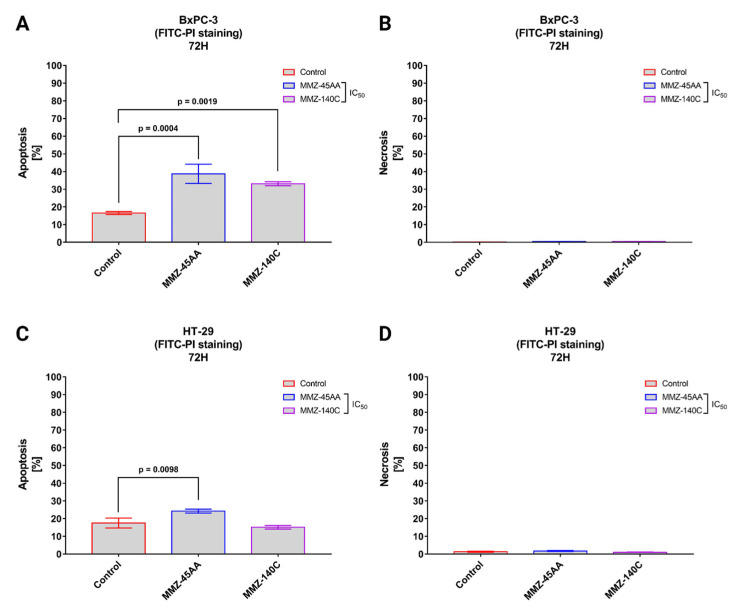
Apoptosis and necrosis induction in BxPC-3 cells (**A**,**B**) and HT-29 cells (**C**,**D**) treated with IC_50_ concentrations of **MMZ-45AA** and **MMZ-140C** compounds measured with Annexin V FITC following 72 h incubation time. Data are presented as mean percentage of apoptotic cells (early and late apoptotic) or necrotic cells ± SD values. The differences between the experimental samples and (untreated) control were evaluated by the ANOVA test followed by Tukey’s test (*p* < 0.05); N = 200.

Flow cytometry with Annexin V conjugated with fluorescein isothiocyanate (FITC) and propidium iodide (PI) is a popular approach for determining cell apoptosis and necrosis. Annexin V is a protein with a high affinity for phosphatidylserine, which is found on the outer membrane surface of apoptotic cells. PI is a fluorescent dye that stains the DNA of necrotic cells with damaged plasma membranes [30]. Similarly to AO/EB staining, we used **MMZ-45AA** and **MM-140C** compounds to further assess apoptosis/necrosis induction in BxPC-3 cells (Figure 7A,B) and HT-29 (Figure 7C,D) after exposure to the compounds in their respective IC_50_ concentrations.

In BxPC-3 cells (Figure 7A), a statistically significant (*p* = 0.0004) increase in apoptotic cell fraction was observed following treatment of cells with IC_50_ concentration of **MMZ-45AA** (% of apoptotic cells = 38.7 ± 5.43%) and **MMZ-140C** (% of apoptotic cells = 33.1 ± 1.12%; *p* = 0.0019) compared with control cells (% of apoptotic cell = 16.5 ± 0.87%). No statistically significant increase (*p* < 0.05) in the necrotic fraction was observed following the treatment of BxPC-3 cells with the two **MMZ** compounds used in IC_50_ concentrations (Figure 7B).

In HT-29 cells (Figure 7C), a statistically significant (*p* = 0.0098) increase in apoptotic cell fraction was observed following treatment of cells with IC_50_ concentration of **MMZ-45AA** (% of apoptotic cells = 24.23 ± 1.1%) compared with control cells (% of apoptotic cell = 17.5 ± 2.8%). No statistically significant increase (*p* = 0.3233) in the number of apoptotic cells was observed following treatment with the **MMZ-140C** compound used in IC_50_ concentration (% of apoptotic cells = 15.13 ± 1%). No statistically significant increase (*p* < 0.05) in the necrotic fraction was observed following the treatment of HT-29 cells with the two **MMZ** compounds used in IC_50_ concentrations (Figure 7D). The final solvent concentration of DMSO was < 0.5% *v/v* in all experimental samples.

### 2.3. Computational Studies

#### 2.3.1. Drug Likeness and ADMET

The processes of chemical absorption, distribution, metabolism, and excretion, collectively referred to as ADMET, play an important role in the discovery and development of new drugs. Not only should a high-quality drug candidate demonstrate adequate efficacy against the therapeutic target, but it should also demonstrate optimal ADMET characteristics at the therapeutic dose. As a result, a significant number of in silico models are being built to make predictions regarding the ADMET properties of new drugs [31]. The prediction of whether or not a biological target can be drugged and the drug-likeness of possible therapeutic agents can be improved by the use of computational methods. The concept of drug-likeness has advanced significantly over the years, taking into account the structural, physicochemical, biochemical, pharmacokinetic, and toxicity attributes of chemical agents. The comprehension of these characteristics has turned into an indispensable aspect of the drug discovery process and made it possible to make precise selections of hits that are appropriate beginning points for the determination of new clinical candidates. The Lipiński rule of five emerged as the analysis of the drug-likeness of compounds based on lipophilicity, molecular weight, and counts of hydrogen bond acceptors and hydrogen bond donors of agents. The Lipiński rule of five is one of the first principles that was proposed for estimating the drug-likeness of compounds. According to Lipinski, to succeed in Phase I clinical trials, a compound should have a molecular weight that is not greater than 500 Da, a log P that is not greater than 5, and no more than 5 moieties capable of acting as hydrogen-bond donors and no more than 10 hydrogen-bond acceptors [32].

The compounds underwent in silico ADMET analysis, which focused solely on their physicochemical properties derived from computationally calculated two-dimensional structural features. The presence of isomeric forms in a particular ligand is attributed to variations in its three-dimensional structural arrangement. Consequently, conducting computational ADMET analysis on isomeric ligands is impractical, as it yields similar/the same outcomes for two distinct isomers, regardless of their structural differences. Therefore, we conducted ADMET analysis on the compounds using *SS*/*RR* isomeric two-dimensional input. ADMET and drug-likeness properties of the investigated compounds **MMZ-33**, **MMZ-39**, **MMZ-45**, **MMZ-140**, **MMZ-147**, and **MMZ-167** are presented in the following section and reveal that **MMZ** compounds follow Lipinski’s rule of five (with one violation in the case of **MMZ-45** related to MLOGP > 4.15). The physicochemical properties obtained for the compounds are tabulated in Table 1.

The pharmacokinetic properties of the investigated **MMZ** compounds are shown in Table 2, including the permeability across the blood–brain barrier (BBB) and gastrointestinal (GI) absorption, together with toxicological effects (hepatotoxicity, cardiotoxicity, and cytochrome P450 (CYP) inhibition) predicted by pkCSM [33], SwissADME [34], and PreADMET [35] online servers, accessed on 26 February 2023.

The synthesized **MMZ** compounds exhibited high GI absorption. Ambiguous prediction results were obtained for CYP inhibitory effects for **MMZ-33**, **MMZ-39**, **MMZ-140**, and **MMZ-147** compounds. In contrast, predictions of inhibitory effects on CYP enzymes have clearly shown that **MMZ-45** and **MMZ-167** may inhibit CYP2D6 and CYP3A4 enzymes. Human CYP enzymes play a significant role in the process of drug detoxification as well as cellular metabolism and homeostasis. In humans, one or more members of the CYP family are accountable for almost 80% of the oxidative metabolism, and CYP2D6 and CYP3A4 enzymes are responsible for the metabolism of more than 50% of the drugs that are now available on the market. CYPs can alter pharmacological responses in a variety of different ways, some of which include the influence they have on the elimination, safety, bioavailability, and resistance to drugs [36].

The blood–brain barrier, often known as the BBB, is the protective measure that separates the tissues of the brain from the substances that are flowing in the circulatory system in the blood. It is also a diffusion barrier that permits the penetrance of water and small, lipophilic molecules into the brain under their concentration gradients [37]. To create drugs that are both effective and efficient, it is necessary to have a solid understanding of how drug molecules interact with the BBB [38]. Among **MMZ** compounds, **MMZ-33**, **-39**, **-45**, and **-140** were found to have physicochemical properties that allow their BBB permeability.

P-glycoprotein, also known as P-gp, is a type of ATP-driven transmembrane transporter that is capable of exporting from the cell a wide variety of hydrophobic molecules that are structurally distinct and functionally unrelated. The phenomenon known as MDR, which is frequently linked to an overexpression of P-gp, has been suggested as a major obstacle in the development of successful chemotherapeutic treatments for a variety of malignancies. By reducing the amount of medicine that is absorbed through the intestinal tract, drug efflux that is mediated by P-gp contributes to a decrease in the oral bioavailability of the drug [39]. **MMZ-140** and **MMZ-147** compounds were found not to act as P-gp substrates.

The Ames test is a bacterial bioassay that is performed in vitro to determine the mutagenicity of a wide range of environmental carcinogens and toxins. The Ames test is used to identify revert mutations that are present in the bacterial strains, but it can also be used to detect the mutagenicity of various substances including drugs that are easily solubilized in a liquid suspension [40]. Only **MMZ-33** was predicted to lack the mutagenic potential in the Ames test, as evidenced by pkCSM and PreADMET online servers.

Cardiotoxicity is a crucial side-effect related with the use of drugs. It may result from the inhibition of the potassium ion channel of the human ether-a-go-go-related gene (hERG) that potentially contributes to the development of long QT syndrome (LQTS) and heart failure [41]. Low and medium risk of cardiotoxicity was predicted by PreADMET software for **MMZ-140** and **MMZ-39,** respectively. Ambiguous predictions were made for **MMZ-33**, **-45**, **-147**, and **167**.

The liver is accountable for a wide variety of functions, some of which include the detoxification of xenobiotics, the synthesis of proteins, the synthesis and storage of glucose, and others. Because of its location downstream from the gastrointestinal tract, the organ can facilitate the “first-pass” clearance of chemicals and medications that are consumed orally. Because hepatocytes have a great capacity for biotransformation, they are also effective at facilitating the production of reactive metabolites, which can lead to damage in the liver [42]. In most instances, hepatotoxicity is identified during later phases of drug development, either during human trials or animal toxicity tests. Even though hepatotoxicity seldom causes drug development to be halted during the preclinical stage, the liver is the organ that is most frequently targeted by drug candidates during animal toxicity tests. In contrast to the toxicity that can be caused in other target organs, liver toxicity is typically reversible and can be monitored in humans through the use of sensitive serum enzyme testing. Therefore, in many instances, a substance that was discovered to be hepatotoxic in an animal species will be evaluated in humans to determine whether or not it has the potential to be hepatotoxic. When medicine has significant therapeutic promise, it is possible that liver damage in humans can be tolerated. In this context, mechanistic investigations are necessary for determining the level of danger posed to humans and, in some instances, for locating protective agents [43]. All **MMZ** compounds were predicted to be hepatotoxic, which contrasts with the Protox-II webserver prediction in which the compounds were classified as inactive for liver toxicity.

The major toxic effects, including hepatoxicity, immunotoxicity, mutagenicity, etc. for the **MMZ** compounds, were additionally predicted by using the Protox-II webserver (http://tox.charite.de/protox_II, accessed on 26 February 2023) [44,45], as shown in Table 3.

Protox-II predictions indicate that **MMZ** compounds do not exhibit hepatotoxic, immunotoxic, or mutagenic properties. This is opposed to the results of pkCSM/PreADMET. However, the software used for the toxicity predictions often use a different set of chemical structures and models for the prediction; therefore, the results may differ. Nonetheless, it is essential to determine whether or not certain substances harm the immune system. Immunotoxicity caused by the medications is a primary contributor to the morbidity and death experienced by patients undergoing therapy. A drug’s effects on the immune system can include immunosuppression, immunostimulation, hypersensitivity, or autoimmunity [46]. Furthermore, we have explored the effect of **MMZ** compounds on the stress response pathways, including the activation of the antioxidant response element (ARE), heat shock response (HSE), cellular tumor antigen p53 (TP53), disruption of mitochondrial membrane potential (MMP), and induction of genotoxicity (ATPase family AAA domain-containing protein 5; ATAD5). In the in silico predictions, **MMZ** compounds were found to not affect these pathways.

#### 2.3.2. Density Functional Theory (DFT) Calculations

The majority of compounds examined in this research comprised *S*,*S*/*R*,*R* enantiomers. This observation, coupled with the enhanced cytotoxicity exhibited by these enantiomeric compounds (specifically, **MMZ-45AA** and **-147B** compounds compared to **MMZ-45B** and **-147CE** derivatives), served as a driving force to explore these forms in subsequent in silico investigations.

The tendency of a molecule in donating and accepting electrons can be estimated using the highest occupied molecular orbital (HOMO) and lowest occupied molecular orbital (LUMO) energy values. The values for these frontier molecular orbital (HOMO, LUMO) and HOMO-LUMO energy gap (HLG) provide information on chemical reactivity, kinetic, structural stability, and electronic properties, and also fetch the most reactive position of a molecule [47]. All of the molecules studied showed similar HOMO, LUMO, and energy gap values, suggesting similar reactivity of the compounds as evidenced by the biological evaluation. The low energy gap value (0.15 eV) obtained denotes the high reactivity, polarizability, and biological activity of the studied molecules [48]. The calculated HOMO and LUMO values were found to be in the range −0.21 to −0.20 eV and 0.05 to −0.04 eV, respectively (Table 4).

The molecular orbital distribution of the molecules is depicted in Supplementary Figure S11 (SM1.pdf file). Interestingly, all the molecules showed the HOMO and LUMO distribution in the naphthalene group, which represents the high reactivity region in the molecules.

**Figure 8:**
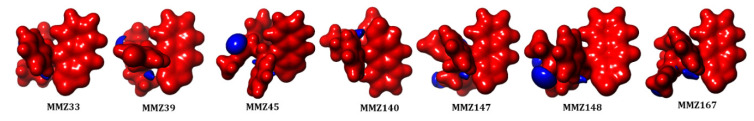
Molecular electrostatic potential (MESP) analysis of **MMZ** compounds.

In addition, molecular electrostatic potential (MESP) analysis was carried out to explore the reactivity and molecular bonding patterns in the compounds. Insights into charge distributions around the molecular surface provided by MESP can be used to identify areas that are vulnerable to electrophilic or nucleophilic attacks during enzymatic processes. The results of molecular docking in conjunction with the electronic characteristics of drug molecules as determined by DFT can also be used to predict noncovalent bonding, such as van der Waals interactions and hydrogen bonds [49]. The red (negative) and blue (positive) represent the electrophilic and nucleophilic reactive sites in the molecules (Figure 8). All the molecules are highly negative and a small portion of positive ESP is located in the oxygen atoms in the 2-(methyl-3-phenylpropanoate) group.

#### 2.3.3. Molecular Target Prediction

**Figure 9:**
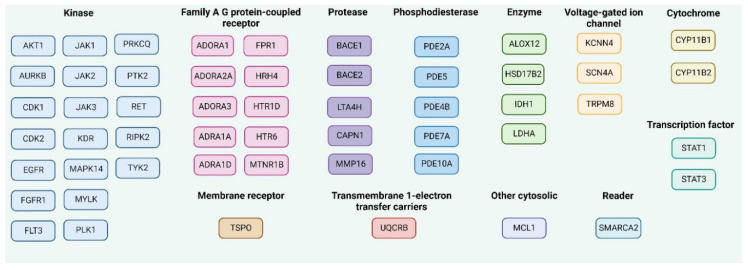
The predicted molecular targets for **MMZ** compounds in Homo Sapiens as the selected organism model using SwissTargetPrediction, accessed on 26 February 2023 and ordered given the protein function. The common targets for **MMZ** compounds are mentioned as one target. Created with Biorender.com.

In addition to the good ADMET profile, a potential drug candidate should demonstrate adequate efficacy against the specifically determined therapeutic target or targets. The knowledge about the chemical structure of the chemical compound can be used for the prediction of the molecular targets for the promising chemical entity [32]. The key fifteen macromolecular targets for each **MMZ** compound that are supposed to interact were predicted by the SwissTargetPrediction web server (http://www.swisstargetprediction.ch/, accessed on 26 February 2023) [50,51], as shown in Figure 9.

Additionally, the SuperPred 3.0 webserver (https://prediction.charite.de/subpages/target_prediction.php, accessed on 26 February 2023), was used for the prediction of possible molecular targets for **MMZ** compounds. We chose the results with the highest probability of being a target (*p* > 80%). Additionally, model accuracies, ChEMBL-IDs, and Protein Data Bank (PDB) codes are shown in Supplementary Table S2 (SM1.pdf file).

#### 2.3.4. Molecular Docking

Molecular docking was performed for targets predicted with the SwissTargetPrediction and SuperPred 3 webservers with available PDB structures (https://www.rcsb.org/, accessed on 26 February 2023) as follows: the generated macromolecular target, together with the native ligands and **MMZ** compounds, were employed by Autogrid software (https://www.auto-grid.com/, accessed on 26 February 2023) to build map files for various atoms of the protein target as well as ligands to run docking analysis. The docking protocols for each of the macromolecular targets that were used in this study were successfully validated by examining the overlay conformation and chemical similarity of the reported native ligand complexed with the bioactive conformation of the investigated target macromolecule. This comparison was carried out to ensure that the docking protocols were accurate representations for the interaction of the native ligand with the protein. After the docking parameters were validated by using the abovementioned parameters, analogous settings were used to run the simulation studies of **MMZ** compounds with predicted targets. The observed docking findings are summarized in Supplementary Table S3 (SM1.pdf file). The compound **MMZ-45** exhibited the best binding properties with ADORA1 (PDBid: 6D9H), CDK1 (PDBid: 2FVD), CDK2 (PDBid: 6GU6), CK (PDBid: 6TLS), NFKB1 (PDBid: 1SVC), PLK1 (PDBid: 3FC2), and TRIM24 (PDBid: 4YBM). For full protein names, see the Abbreviations section.

#### 2.3.5. Molecular Dynamics Simulation

**Figure 10:**
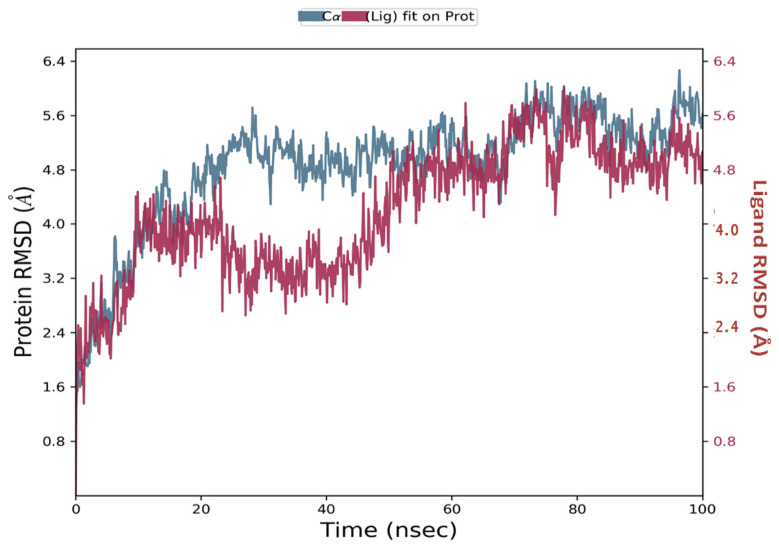
Root-mean-square deviation (RMSD) observed for the macromolecular complex of **MMZ-45** complexed within the macromolecular backbone of adenosine A1 (ADORA1) receptor during MD simulation of 100 ns.

Simulations of molecular dynamics revealed that the macromolecular complexes of ligands **MMZ-45** against adenosine A1 receptor (ADORA1) were found to be most stable throughout the simulation time, concluding that the ligand **MMZ-45** was supposed to be a potent anticancer agent and its therapeutic effect is executed via targeting ADORA1 enzyme. The target protein ADORA1 has 288 residues distributed in a macromolecular chain consisting of 2276 heavy atoms out of a total of 4703 atoms. The macromolecular target has only 58.7% beta strands constituting the secondary structure of the protein that remains conserved throughout the simulation process. Ligand **MMZ-45** possesses 31 heavy atoms out of a total of 56 atoms. Dynamic simulation of the **MMZ-45** macromolecular complex against the ADORA1 target clearly showed that the root-mean-square deviation (RMSD) for the fluctuation of the protein backbone was between 1.8 and 5.8 Å, which is well within the acceptable range. The macromolecular backbone required two conformational changes within the initial 20 ns to achieve the most stabilized conformation, which was maintained throughout the remaining simulation. Similarly, to achieve a stabilized conformation, the ligand **MMZ-45** showed some initial fluctuations until 15 ns and then maintained the same throughout the simulation with the RMSD ranging between 4.0 and 5.6 Å. Observed RMSD for the macromolecular complex of **MMZ-45** with the ADORA1 receptor is depicted in Figure 10.

**Figure 11:**
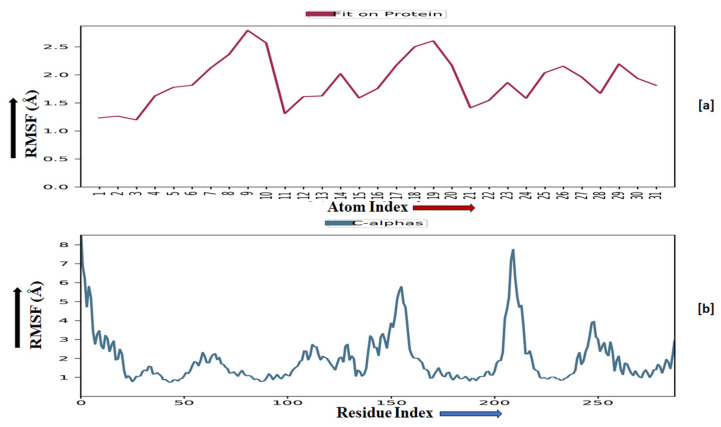
Root-mean-square fluctuation (RMSF) observed for the macromolecular complex of **MMZ-45** (**a**) together with the macromolecular backbone of the adenosine A1 (ADORA1) receptor (**b**) during MD simulation of 100 ns.

The root-mean-square fluctuation (RMSF) of the macromolecular backbone was found to be well within the range for the ADORA1 receptor, which was found to be 1–3 Å, except for some residues having higher fluctuations. The RMSF for the complexed ligand **MMZ-45** was found to be within 2–3 Å throughout the simulation. RMSF observed for **MMZ-45** and the macromolecular backbone of ADORA1 receptor during 100 ns MD simulation is depicted in Figure 11.

**Figure 12:**
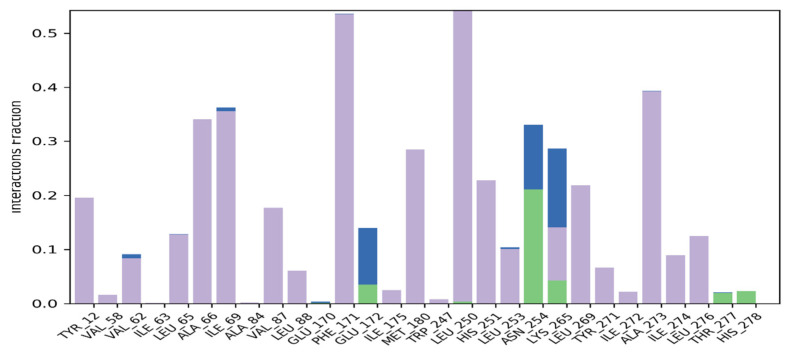
Ligand-receptor contacts for **MMZ-45** and adenosine A1 (ADORA1) receptor observed during MD simulation of 100 ns. Purple—hydrophobic interaction; green—hydrogen bonds; blue—water bridges.

Macromolecular residues such as Tyr12, Val62, Leu65, Ala66, Ile69, Val87, Leu88, Phe171, Met180, Leu250, His251, Leu253, Leu269, Tyr271, Ala273, Ile274, and Leu276 were found to be interacting hydrophobically. Residues like Asn254, Thr277, His278, via forming H-bonds and residues like Glu172, and Lys265 were found to be interacting via forming water bridges with the complexed ligand **MMZ-45**. The ligand receptor contacts between **MMZ-45** and ADORA1 receptor observed during 100 ns MD simulation are depicted in Figure 12.

Moreover, sufficient stability was observed for **MMZ-45** and two other enzymes: cyclin-dependent kinase 2 (CDK2) and transcription intermediary factor 1-alpha (TRIM24).

**Figure 13:**
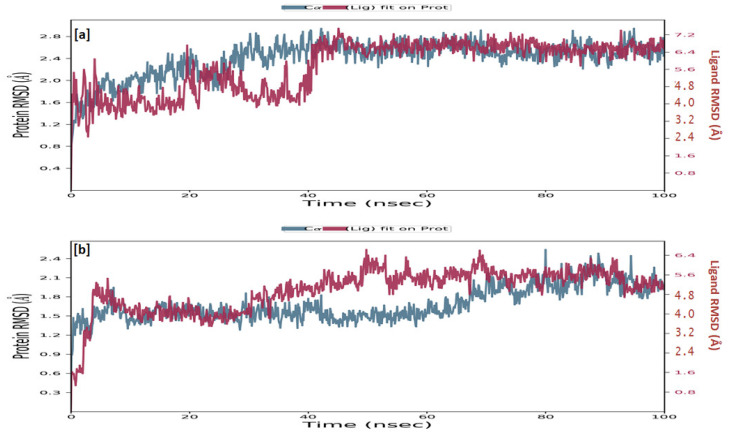
Root-mean-square deviation (RMSD) observed for the macromolecular complex consisting of **MMZ-45** complexed within the macromolecular backbone of transcription intermediary factor 1-alpha (TRIM24) (**a**) and cyclin-dependent kinase 2 (CDK2) (**b**) observed during 100 ns MD simulation.

**Figure 14:**
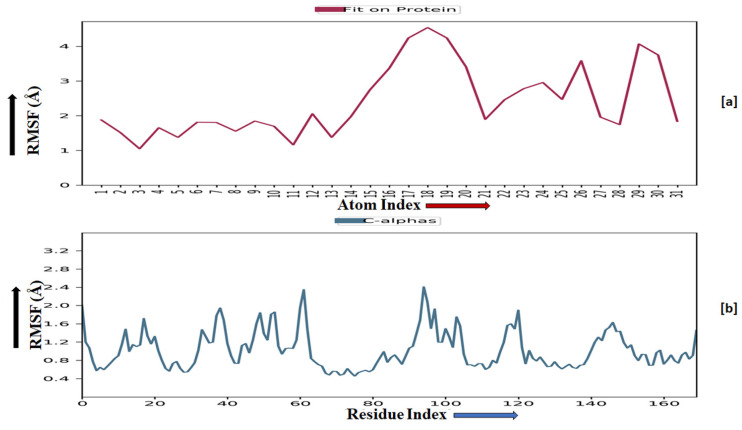
Root-mean-square fluctuation (RMSF) observed for the macromolecular complex of **MMZ-45** (**a**) and the macromolecular backbone of transcription intermediary factor 1-alpha (TRIM24) protein (**b**) observed during 100 ns MD simulation.

TRIM24 consists of 170 residues and forms a macromolecular chain with a total of 2278 atoms, of which 1384 are heavy. The secondary structure analysis indicated that the protein predominantly comprises 30% alpha helices and 1% beta strands, and this secondary structure conformation remained conserved during the simulation process. **MMZ-45** contains 56 atoms, with 31 of them being heavy atoms. The dynamic simulation of the macromolecular complex formed by **MMZ-45** and the TRIM24 target demonstrated that RMSD for the protein backbone fluctuations ranged from 1.2 to 2.5 Å, which falls within an acceptable range (Figure 13). Importantly, the macromolecular backbone exhibited a stable conformation throughout the simulation period. Similarly, the ligand **MMZ-45** initially showed some fluctuations at 20 and 40 ns, but achieved a stabilized conformation thereafter, maintaining this conformation throughout the simulation with an RMSD ranging between 6.0 and 7.0 Å. The RMSF analysis of the TRIM24 macromolecular backbone showed values within the range 0.5 to 2.0 Å for most residues, with a few residues exhibiting slightly higher fluctuations. As for the complexed ligand **MMZ-45**, the RMSF ranged from 1 to 4 Å during the entire simulation (Figure 14).

**Figure 15:**
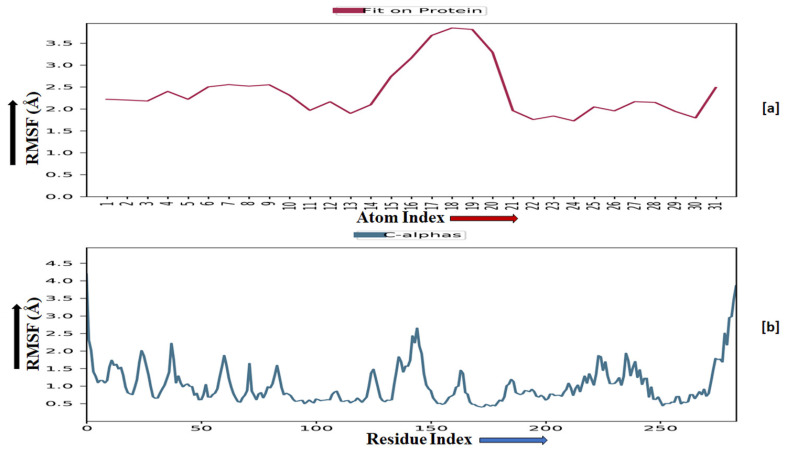
Root-mean-square fluctuation (RMSF) observed for the macromolecular complex of **MMZ-45** (**a**) and the macromolecular backbone of cyclin-dependent kinase 2 (CDK2) (**b**) observed during 100 ns MD simulation.

The CDK2 enzyme is composed of 284 residues, forming a macromolecular chain comprising a total of 4645 atoms, with 2292 of them being heavy atoms. The secondary structure analysis revealed that the protein consists of 23.4% alpha helices and 13.76% beta strands, and this secondary structure conformation remained conserved throughout the simulation process. The dynamic simulation of the macromolecular complex involving **MMZ-45** and the CDK2 target demonstrated that the RMSD for the protein backbone fluctuations fell within the range 1.2 to 2.4 Å, which is considered acceptable (Figure 13). Initially, the ligand **MMZ-45** exhibited some fluctuations until 10 ns, after which it attained a stabilized conformation that was maintained throughout the simulation, with the RMSD ranging from 4.0 to 6.0 Å (Figure 13). RMSF analysis of the CDK2 macromolecular backbone revealed values well within the range 0.5 to 2 Å, except for a few terminal residues exhibiting higher fluctuations. In contrast, the RMSF for the ligand **MMZ-45** in complex with CDK2 remained within the range 2 to 3 Å throughout the simulation (Figure 15). 

**Figure 16:**
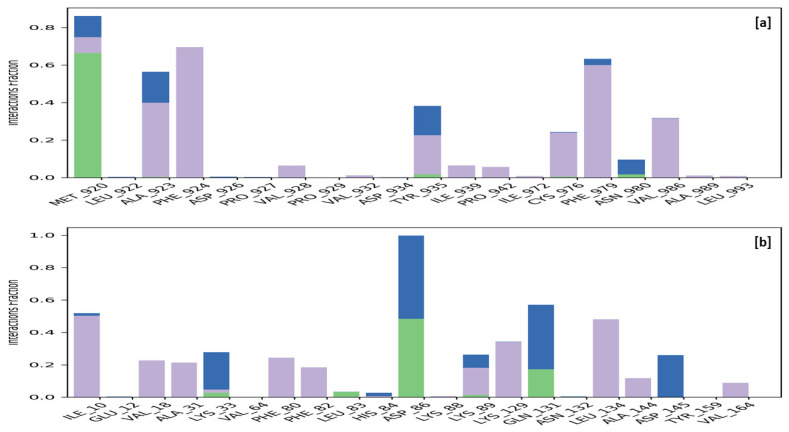
Ligand-receptor contacts for **MMZ-45** with transcription intermediary factor 1-alpha (TRIM24) (**a**) and **MMZ-45** with cyclin-dependent kinase 2 (CDK2) (**b**) observed during 100 ns MD simulation. Purple—hydrophobic interaction; green—hydrogen bonds; blue—water bridges.

Specific macromolecular residues of TRIM24, including Ala923, Phe924, Val928, Ile939, Pro942, Cys976, Phe979, and Val986, were found to interact hydrophobically, while residue Met920 formed hydrogen bonds with the ligand **MMZ-45**. Additionally, residues Met920, Ala923, Tyr935, and Asn980 were involved in water-bridge interactions with the ligand **MMZ-45**. For CDK2 enzyme macromolecular residues, such as Ile10, Val18, Ala31, Phe80, Phe82, Lys89, Lys129, Leu134, Ala144, and Val164, were found to be interacting hydrophobically; residues like Leu83, Asp86, and Gln131, via forming H-bonds and residues like Lys33, Asp86, Lys89, Gln131, and Asp145, were found to be interacting via forming water bridges with the complexed ligand **MMZ-45** (Figure 16).

These results provide insights into the stability and interactions within the macromolecular complex, highlighting the conformational behavior of both the protein backbone and the ligand **MMZ-45** during the dynamic simulation. Given the limited information on ADORA1 in cancer pathophysiology, the observed anticancer effects can be attributed to the inhibition of the other two targets (TRIM24 and CDK2).

#### 2.3.6. Prime MM-GBSA Analysis

The binding strength between protein and ligand molecule was estimated using Prime MM-GBSA analysis. The calculated free energy of binding for the protein–ligand complexes was in the range −66.73 to −23.71 kcal/mol (Table 5). The binding free energy of **MMZ-45** complexed CDK2 (PDB id: 2FVD) (−66.73 kcal/mol) and **MMZ-45** complexed with ADORA1 (PDB id: 6D9H) (−51.57 kcal/mol) had the most negative free energy of binding, which signifies the stronger binding of ligands into the active site of the respective proteins. Other complexes also showed reasonable free energy of binding with the targets. Van der Waals, polar, and nonpolar solvation favors the binding of ligands into the binding site of the respective proteins. The result confirmed the contribution of various free energies of binding in attaining stable conformation, and also showed that the complexes are thermodynamically stable.

## 3. Discussion

The investigated **MMZ** compounds exhibited potent cytotoxic activity with IC_50_ values between 30.15 ± 9.39 and 66.19 ± 7.36 µM following 24 h treatment of BxPC-3 cells, and a cytotoxicity between 31.78 ± 3.93 and 111.5 ± 2.12 µM for HT-29 cells. In contrast, 72 h incubation with tested derivatives resulted in the cytotoxicity range 13.26 to 54.55 μM in BxPC-3 cells and 11.55 to 58.11 μM in the HT-29 cell line. At the same time, the compounds did not exhibit pronounced cytotoxic potential and did not reduce normal cell (WI-38) viability to a level below 70%, except for **MMZ-39AA**, **MMZ-147B**, and **MM-147CE** when used in respective IC_50_ values obtained for cancer cells.

Furthermore, for the first time, we report the proapoptotic potential of two **MMZ** derivatives (**MMZ-45AA** and **MMZ-140C**) as estimated by the exposition of phosphatidylserine on the surface of cancer cells and morphological changes observed during staining with AO/EB. A more profound apoptotic response was observed for BxPC-3 cells, for example, following 72 h incubation of BxPC-3 cells with **MMZ-45AA** used in IC_50_ concentration % of apoptotic cells = 21.9 ± 3.4% for OA/EB staining and 38.7 ± 5.43% for annexin-FITC staining, compared with HT-29 cells where % of apoptotic cells = 15.93 ± 3% for OA/EB staining and 24.23 ± 1.1% for annexin-FITC staining. Furthermore, the **MMZ-140C** compound induced a statistically significant increase in apoptotic cell fraction (% of apoptotic cells = 33.1 ± 1.12%) only in BxPC-3 cells (as evidenced by annexin-FITC staining). No changes in necrotic cell fractions were observed in any of the experimental samples compared with cells used as the negative control.

Using in silico approaches, we show that the compounds exhibit drug-likeness properties and good pharmacokinetic properties, including high GI absorption and BBB permeability (except for **MMZ-147**). However, their future therapeutical use may be restricted by CYP enzymes’ inhibitory properties and the fact that the compounds may act as substrates of P-gp. Furthermore, DFT and MESP calculations were performed to establish the chemical reactivity of the compounds and show the electrophilic and nucleophilic components of the compounds. The naphthalene group was proposed as the high reactivity region in the molecules examined.

A potential drug candidate should exhibit sufficient efficacy against the explicitly specified therapeutic target or targets, in addition to a favorable ADMET profile. Therefore, we predicted probable molecular targets for **MMZ** compounds by using the SwissTargetPrediction web server (http://www.swisstargetprediction.ch/ or SuperPred 3.0 webserver https://prediction.charite.de/subpages/target_prediction.php, and performed molecular docking investigation to select probable targets for the compounds (software accessed on 26 February 2023). Among the compounds, **MMZ-45** showed the best binding efficiency and stability with ADORA1, CDK2, and TRIM24 in the docking and initial molecular dynamics simulation. Therefore, we performed a 100 ns MD simulation to further reveal the stability of the formed complexes and determine the crucial amino acids involved in associations. The binding strength between protein targets and **MMZ-45** molecule was estimated using Prime MM-GBSA analysis. The calculated free energy of binding for the protein–ligand complexes was in the range −66.73 to −23.71 kcal/mol. The binding free energy of **MMZ-45** complexed CDK2 (PDB id: 2FVD) (−66.73 kcal/mol) an **MMZ-45** complexed with ADORA1 (PDB id: 6D9H) (−51.57 kcal/mol) had the most negative free energy of binding, which signifies a stronger binding of ligands into the active site of the respective proteins.

This study indicates that aminobenzylnaphthols (**MMZ** compounds) obtained by the Betti reaction using methyl derivatives of (*R*)-valine, (*S*)-phenylalanine, (*S*)-proline, (*S*)-glutamic acid, (*S*)-aspartic acid, and (*S*)-leucine exhibit potent cytotoxic and proapoptotic properties and constitute a profound scaffold for anticancer drug discovery. The best cytotoxic properties were observed for Betti bases containing (*S*)-proline and (*S*)-phenylalanine (**MMZ-45AA** and **-140C**), with an aromatic or heterocyclic fragment present in the amino acid part. Aliphatic amino acid derivatives in these tests were characterized by weaker cytotoxic properties. The compounds exhibited comparable cytotoxic activity to 5-Fluorouracil used as a positive control in our study. Moreover, the use of normal human fibroblasts allowed us to show preferential activity of the tested compounds toward cancer cells. The resulting antitumor activity may, in turn, be derived from the inhibition of important molecular targets involved in cancer pathogenesis, including ADORA1, CDK2, and TRIM24. Future studies will elucidate the exact molecular mechanisms in the anticancer activity of these compounds that could explain potential selectivity toward cancer cells.

## 4. Materials and Methods

### 4.1. Chemical Studies

#### Synthesis of Betti Bases (MMZs)

Commercially available chemicals used in this work were purchased from Sigma-Aldrich and were used as supplied, without additional purification. NMR spectra were recorded in CDCl_3_ on a Bruker Avance III (600 MHz for 1H NMR, 150 MHz for 13C NMR); coupling constants are reported in hertz (Hz). The chemical shift values were expressed in ppm (part per million) with tetramethylsilane (TMS) as an internal reference. The rotations were measured using an Anton Paar MCP 500 polarimeter. Melting points measured on the DigiMelt apparatus were uncorrected. Chromatographic purification of compounds was achieved with 230−400 mesh size silica gel. The progress of reactions was monitored by silica gel thin-layer chromatography plates (Merck TLC Silicagel 60 F254).

Aminoacids methyl ester (1.46 mmol, 1.2 eq.) was added to benzaldehyde or *p-*chlorobenzaldehyde (1.27 mmol, 1.05 eq.) and stirred for 10 min at room temperature under an argon atmosphere. 2-Naphthol (174 mg, 1.21 mmol, 1 eq.) was added and the mixture was heated to 60 °C for two days. The progress of the reaction was monitored by silica gel thin-layer chromatography plates (*n*-hexane/ethyl acetate 4:1). The crude reaction mixture was purified first by chromatography (silica gel, eluent n-hexane/ethyl acetate 4:1), followed by crystallization (ethanol) to obtain the product.

***(S,S) and (R,R)-methyl ((2-hydroxynaphthalen-1-yl)(phenyl)methyl)-d-valinate (MMZ-33D)***

Colorless solid. Yield 23%. R_f_ = 0.49; Melting point: 74.5–76.5 °C {Lit.:^22^ m.p. = 146–148 °C}; [α]_D_^20^ = 106.34 (*c* 0.50, CHCl_3_) {Lit.:^22^ [α]_D_^20^ = 428.0 (*c* 0.36, CHCl_3_)}; ^1^H NMR (CDCl_3_) δ: 0.97 (d, 3H, *J* = 6.9, CH_3_), 0.98 (d, 3H, *J* = 6.9, CH_3_), 2.04–2.12 (m, 1H, C*H*(CH_3_)_2_), 2.68 (d, 1H, *J* = 12.8 Hz, NH), 3.33 (dd, 1H, *J* = 12.7, 5.3 Hz, CHCO), 3.79 (s, 3H, OCH_3_), 5.58 (s, 1H, CHAr_2_), 7.16 (d, 1H, *J* = 8.8 Hz, H_Ar_), 7.22–7.27 (m, 2H, H_Ar_), 7.29–7.34 (m, 3H, H_Ar_), 7.43 (d, 2H, *J* = 7.5 Hz, H_Ar_), 7.59 (d, 1H, *J* = 8.6 Hz, H_Ar_), 7.74 (d, 2H, *J* = 9.1 Hz, H_Ar_), 12.49 (s, 1H, OH). Spectral data matched that reported by Bedekar [52].

***(S,S) and (R,R)-methyl ((4-chlorophenyl)(2-hydroxynaphthalen-1-yl)methyl)-d-valinate (MMZ-39AA)***

Colorless solid. Yield 48%. R_f_ = 0.65; Melting point: 64–65.5 °C {Lit.:^21^ m.p. = 119–121 °C}; [α]_D_^20^ = −63.32 (*c* 0.52, CHCl_3_) {Lit.:^21^ [α]_D_^20^ = 105.0 (*c* 0.9, CHCl_3_)}; ^1^H NMR (CDCl_3_) δ: 0.96–1.0 (m, 6H, 2xCH_3_), 2.05–2.13 (m, 1H, C*H*(CH_3_)_2_), 2.63 (d, 1H, *J* = 12.8 Hz, NH), 3.34 (dd, 1H, *J* = 12.3, 5.1 Hz, CHCO), 3.79 (s, 3H, OCH_3_), 5.55 (s, 1H, CHAr_2_), 7.16 (d, 1H, *J* = 8.9 Hz, H_Ar_), 7.26–7.29 (m, 3H, H_Ar_), 7.31–7.35 (m, 1H, H_Ar_), 7.37 (d, 2H, *J* = 8.5 Hz, H_Ar_), 7.54 (d, 1H, *J* = 8.6 Hz, H_Ar_), 7.75 (d, 2H, *J* = 9.20 Hz, H_Ar_), 12.39 (s, 1H, OH). Spectral data matched that reported by Cardellicchio [53].

***(S,S) and (R,R)-methyl ((2-hydroxynaphthalen-1-yl)(phenyl)methyl)-l-phenylalaninate (MMZ-45AA)***

Colorless solid. Yield 38%. R_f_ = 0.67; Melting point: 114–115.5 °C; [α]_D_^20^ = 2.31 (*c* 0.39, CHCl_3_); ^1^H NMR (CDCl_3_) δ: 2.67 (d, 1H, *J* = 12.4 Hz, NH), 3.06 (dd, 1H, *J* = 13.7, 6.8, CH_2_Ar), 3.14 (dd, 1H, *J* = 13.7, 5.4, CH_2_Ar), 3.71 (s, 3H, OCH_3_), 3.75–3.82 (m, 1H, CHCO), 5.63 (s, 1H, CHAr_2_), 7.08 (dd, 2H, *J* = 8.1, 1.4 Hz, H_Ar_), 7.15 (d, 1H, *J* = 8.9 Hz, H_Ar_), 7.21–7.32 (m, 8H, H_Ar_), 7.40 (d, 2H, *J* = 7.3 Hz, H_Ar_), 7.57 (d, 1H, *J* = 8.6 Hz, H_Ar_), 7.74 (d, 2H, *J* = 8.6 Hz, H_Ar_), 12.61 (s, 1H, OH); ^13^C NMR (CDCl_3_) δ: 39.6 (CH_2_Ar), 52.2 (OCH_3_), 60.7 (CHCO), 61.9 (CHAr_2_), 112.5 120.2, 121.2, 122.7, 126.7, 127.3, 128.1, 128.3, 128.8, 128.9, 129.0, 129.2, 129.4, 130.1, 132.9, 135.7, 140.5, 156.9 (C_Ar_), 174.1 (CO); elementar analysis: C_27_H_25_NO_3_ (411.50 g/mol) calculated: C% 78.81, H% 6.12, N% 3.40; found: C% 78.80, H% 6.10, N% 3.60.

***(S,R) and (R,S)-methyl ((2-hydroxynaphthalen-1-yl)(phenyl)methyl)-l-phenylalaninate (MMZ-45B)***

Colorless solid. Yield 22%. R_f_ = 0.52; Melting point: 73.5–75.0 °C; [α]_D_^20^ = −6.74 (*c* 0.34, CHCl_3_); ^1^H NMR (CDCl_3_) δ: 2.67 (bs, 1H, NH), 3.01 (dd, 1H, *J* = 13.8, 7.3, CH_2_Ar), 3.14 (dd, 1H, *J* = 13.8, 5.3, CH_2_Ar), 3.66 (dd, 1H, *J* = 7.3, 5.3, CHCO), 3.72 (s, 3H, OCH_3_), 5.83 (s, 1H, CHAr_2_), 7.12 (d, 1H, *J* = 8.9 Hz, H_Ar_), 7.13 (d, 2H, *J* = 7.0 Hz, H_Ar_), 7.18–7.26 (m, 4H, H_Ar_), 7.28–7.32 (m, 3H, H_Ar_), 7.32–7.39 (m, 3H, H_Ar_), 7.66 (d, 1H, *J* = 8.9 Hz, H_Ar_), 7.70 (d, 1H, *J* = 8.0 Hz, H_Ar_), 7.76 (d, 1H, *J* = 8.7 Hz, H_Ar_), 12.10 (s, 1H, OH); ^13^C NMR (CDCl_3_) δ: 38.9 (CH_2_Ar), 52.3 (OCH_3_), 59.3 (CHCO), 60.5 (CHAr_2_), 115.4 120.4, 121.2, 122.7, 126.7, 127.4, 128.2, 128.2, 129.0, 129.1, 129.3, 130.0, 132.2, 136.3, 140.0, 155.9 (C_Ar_), 173.4 (CO); elementar analysis: C_27_H_25_NO_3_ (411.50 g/mol) calculated: C% 78.81, H% 6.12, N% 3.40; found: C% 78.64, H% 53.92, N% 3.15.

***(S,S) and (R,R)-methyl ((2-hydroxynaphthalen-1-yl)(phenyl)methyl)-l-prolinate (MMZ-140C)***

Colorless solid. Yield 33%. R_f_ = 0.38; Melting point: 119.5–120.5 °C; [α]_D_^20^ = 0.63 (*c* 0.32, CHCl_3_); ^1^H NMR (CDCl_3_) δ: 1.91–2.00 (m, 2H, CH), 2.01–2.10 (m, 1H, CH), 2.23–2.31 (m, 1H, CH), 2.60–2.67 (m, 1H, CH), 3.37–3.45 (m, 1H, CH), 3.43 (s, 3H, OCH_3_), 3.60 (dd, 1H, *J* = 9.3, 4.7 Hz, CHN), 5.45 (s, 1H, CHAr_2_), 7.20–7.30 (m, 5H, H_Ar_), 7.36–7.40 (m, 1H, H_Ar_), 7.59 (d, 1H, *J* = 7.1 Hz, H_Ar_), 7.72 (d, 1H, *J* = 9.3 Hz, H_Ar_), 7.73 (d, 1H, *J* = 9.0 Hz, H_Ar_), 7.84 (d, 1H, *J* = 8.6 Hz, H_Ar_), 13.22 (s, 1H, OH); ^13^C NMR (CDCl_3_) δ: 24.4 (CH_2_), 30.9 (CH_2_), 38.9 (CH_2_Ar), 52.0 (OCH_3_, CH_2_N), 63.2 (CHN, CHAr_2_), 116.3, 120.2, 121.1, 122.6, 126.5, 128.4, 128.6, 128.8, 129.0, 129.8, 130.0, 132.0, 139.7, 155.4 (C_Ar_), 174.3 (CO); elementar analysis: C_23_H_23_NO_3_ (361.44 g/mol) calculated: C% 76.43, H% 6.41, N% 3.88; found: C% 76.42, H% 6.67, N% 3.86.

***(S,S) and (R,R)-dimethyl ((2-hydroxynaphthalen-1-yl)(phenyl)methyl)-l-aspartate (MMZ-147B)***

Colorless solid. Yield 57%. R_f_ = 0.35; Melting point: 153.0–154.5 °C; [α]_D_^20^ = 9.79 (*c* 0.42, CHCl_3_); ^1^H NMR (CDCl_3_) δ: 2.86–2.94 (m, 2H, CH_2_), 3.22 (d, 1H, *J* = 11.3 Hz, NH), 3.66 (s, 3H, OCH_3_), 3.67–3.73 (m, 1H, CHN), 3.81 (s, 3H, OCH_3_), 5.93 (s, 1H, CHAr_2_), 7.16 (d, 1H, *J* = 8.9 Hz, H_Ar_), 7.23–7.28 (m, 1H, H_Ar_), 7.29–7.35 (m, 3H, H_Ar_), 7.50 (d, 2H, *J* = 7.3 Hz, H_Ar_), 7.65 (d, 1H, *J* = 8.6 Hz, H_Ar_), 7.73 (d, 2H, *J* = 8.7 Hz, H_Ar_), 12.33 (s, 1H, OH); ^13^C NMR (CDCl_3_) δ: 37.5 (CH_2_), 52.1 (OCH_3_), 52.6 (OCH_3_), 55.5 (CHCO), 62.0 (CHAr_2_), 112.5, 120.0, 121.2, 122.7, 126.7, 128.0, 128.2, 128.7, 128.8, 129.1, 130.1, 132.9, 140.2, 156.6 (C_Ar_), 170.9 (CO), 173.0 (CO); elementar analysis: C_23_H_23_NO_5_ (393.44 g/mol) calculated: C% 70.21, H% 5.89, N% 3.56; found: C% 70.02, H% 5.96, N% 3.61.

***(S,R) and (R,S)-dimethyl ((2-hydroxynaphthalen-1-yl)(phenyl)methyl)-l-aspartate (MMZ-147C)***

Colorless solid. Yield 25%. R_f_ = 0.27; Melting point: 119.5–121.0 °C; [α]_D_^20^ = −3.40 (*c* 0.47, CHCl_3_); ^1^H NMR (CDCl_3_) δ: 2.81–2.91 (m, 2H, CH_2_), 3.06 (bs, 1H, NH), 3.68 (s, 3H, OCH_3_), 3.75 (s, 3H, OCH_3_), 3.75–3.79 (m, 1H, CHN), 5.95 (s, 1H, CHAr_2_), 7.15 (d, 1H, *J* = 8.9 Hz, H_Ar_), 7.23–7.26 (m, 2H, H_Ar_), 7.28–7.32 (m, 2H, H_Ar_), 7.35–7.39 (m, 1H, H_Ar_), 7.47 (d, 2H, *J* = 7.4 Hz, H_Ar_), 7.70 (d, 1H, *J* = 8.9 Hz, H_Ar_), 7.72 (d, 1H, *J* = 8.1 Hz, H_Ar_), 7.78 (d, 1H, *J* = 8.7 Hz, H_Ar_), 12.22 (s, 1H, OH); ^13^C NMR (CDCl_3_) δ: 35.2 (CH_2_), 52.2 (OCH_3_), 52.7 (OCH_3_), 54.7 (CHCO), 59.9 (CHAr_2_), 114.2, 120.3, 121.1, 122.7, 126.6, 126.8, 126.9, 128.1, 128.2, 128.3, 128.8, 129.2, 129.3, 130.1, 132.2, 140.0, 156.1 (C_Ar_), 170.9 (CO), 172.2 (CO); elementar analysis: C_23_H_23_NO_5_ (393.44 g/mol) calculated: C% 70.21, H% 5.89, N% 3.56; found: C% 70.07, H% 5.92, N% 3.27.

***(S,S) and (R,R)-dimethyl ((2-hydroxynaphthalen-1-yl)(phenyl)methyl)-l-glutamate (MMZ-148B)***

Colorless solid. Yield 47%. R_f_ = 0.22; [α]_D_^20^ = 27.88 (*c* 0.52, CHCl_3_); ^1^H NMR (CDCl_3_) δ: 2.01–2.15 (m, 2H, CH_2_), 2.44 (t, 1H, *J* = 7.6 Hz, CH_2_CO) 2.75 (d, 1H, *J* =12.0 Hz, NH), 3.50–3.57 (m, 1H, CHN), 3.63 (s, 3H, OCH_3_), 3.78 (s, 3H, OCH_3_), 5.63 (s, 1H, CHAr_2_), 7.16 (d, 1H, *J* = 8.9 Hz, H_Ar_), 7.21–7.27 (m, 2H, H_Ar_), 7.29–7.33 (m, 3H, H_Ar_), 7.44 (d, 2H, *J* = 7.4 Hz, H_Ar_), 7.59 (d, 1H, *J* = 8.6 Hz, H_Ar_), 7.74 (d, 2H, *J* = 8.7 Hz, H_Ar_), 12.39 (s, 1H, OH); ^13^C NMR (CDCl_3_) δ: 28.4 (CH_2_), 30.2 (CH_2_CO), 51.9 (OCH_3_), 52.5 (OCH_3_), 58.9 (CHCO), 61.9 (CHAr_2_), 112.4 120.1, 121.1, 122.8, 126.8, 128.0, 128.4, 128.9, 129.0, 129.2, 130.2, 132.9, 140.4, 156.6 (C_Ar_), 172.8 (CO), 174.5 (CO); elementar analysis: C_24_H_25_NO_5_ (407.47 g/mol) calculated: C% 70.75, H% 6.18, N% 3.44; found: C% 70.80, H% 6.18, N% 3.38.

***(S,R) and (R,S)-dimethyl ((2-hydroxynaphthalen-1-yl)(phenyl)methyl)-l-glutamate (MMZ-148C)***

Colorless oil. Yield 10%. R_f_ = 0.17; [α]_D_^20^ = −15.64 (*c* 0.36, CHCl_3_); ^1^H NMR (CDCl_3_) δ: 0.04–2.17 (m, 2H, CH_2_), 2.41 (t, 1H, *J* = 7.5 Hz, CH_2_CO) 2.77 (bs, 1H, NH), 3.37–3.43 (m, 1H, CHN), 3.63 (s, 3H, OCH_3_), 3.76 (s, 3H, OCH_3_), 5.87 (s, 1H, CHAr_2_), 7.15 (d, 1H, *J* = 8.9 Hz, H_Ar_), 7.22–7.26 (m, 2H, H_Ar_), 7.28–7.32 (m, 2H, H_Ar_), 7.36–7.40 (m, 1H, H_Ar_), 7.49 (d, 2H, *J* = 7.3 Hz, H_Ar_), 7.68 (d, 1H, *J* = 8.9 Hz, H_Ar_), 7.71 (d, 1H, *J* = 8.0 Hz, H_Ar_), 7.82 (d, 1H, *J* = 8.6 Hz, H_Ar_), (12.31 (s, 1H, OH); ^13^C NMR (CDCl_3_) δ: 27.3 (CH_2_), 30.1 (CH_2_CO), 51.9 (OCH_3_), 52.4 (OCH_3_), 57.1 (CHCO), 60.4 (CHAr_2_), 115.3, 129.3, 121.2 122.8, 126.8, 128.3, 128.8, 128.9, 129.2, 130.1, 132.1, 140.0, 155.8 (C_Ar_), 172.8 (CO), 173.5 (CO); elementar analysis: C_24_H_25_NO_5_ (407.47 g/mol) calculated: C% 70.75, H% 6.18, N% 3.44; found: C% 70.64, H% 6.19, N% 3.24.

***(S,S) and (R,R)-methyl ((2-hydroxynaphthalen-1-yl)(phenyl)methyl)-l-leucinate (MMZ-167C)***

Colorless solid. Yield 41%. R_f_ = 0.55; Melting point: 92.5–93.5 °C; [α]_D_^20^ = 52.4 (*c* 0.45, CHCl_3_); ^1^H NMR (CDCl_3_) δ: 0.86 (d, 3H, *J* = 6.9, CH_3_), 0.88 (d, 3H, *J* = 7.0, CH_3_), 1.54–1.63 (m, 2H, CH_2_), 1.72–1.81 (m, 1H, C*H*(CH_3_)_2_), 2.65 (d, 1H, *J* = 12.5 Hz, NH), 3.49–3.55 (m, 1H, CHCO), 3.78 (s, 3H, OCH_3_), 5.61 (s, 1H, CHAr_2_), 7.16 (d, 1H, *J* = 8.9 Hz, H_Ar_), 7.22–7.25 (m, 2H, H_Ar_), 7.27–7.34 (m, 3H, H_Ar_), 7.42 (d, 2H, *J* = 7.4 Hz, H_Ar_), 7.58 (d, 1H, *J* = 8.6 Hz, H_Ar_), 7.72 (d, 1H, *J* = 8.9 Hz, H_Ar_), 7.74 (d, 1H, *J* = 7.7 Hz, H_Ar_), 12.49 (s, 1H, OH),; ^13^C NMR (CDCl_3_) δ: 22.5 (CH_3_), 22.7 (CH_3_), 25.1 (C*H*(CH_3_)_2_)), 43.2 (CH_2_), 52.2 (OCH_3_), 58.5 (CHCO), 61.8 (CHAr_2_), 112.7, 120.1, 121.2, 122.7, 126.7, 128.1, 128.3, 128.9, 129.0, 129.2, 130.2, 133.0, 140.7, 156.9 (C_Ar_), 175.8 (CO); elementar analysis: C_24_H_27_NO_3_ (377.48 g/mol) calculated: C% 76.36, H% 7.21, N% 3.71; found: C% 76.30, H% 7.05, N% 3.60.

### 4.2. Biological Studies

#### 4.2.1. Chemicals

Trypsin-EDTA and RPMI-164 were purchased from Biowest (CytoGen, Zgierz, Poland). Amino acids solution (MEM), acridine orange/ethidium bromide (AO/BE), buffered saline (PBS), 5-Fluorouracil, sodium chloride, penicillin–streptomycin solution stabilized, fetal bovine serum (FBS), dimethyl sulfoxide (DMSO), and MTT 3-(4,5-dimethylthiazol-2-yl)-2,3-diphenyltetrazolium bromide were supplied by Merck/Sigma Aldrich Chemical Co (Burlington, MA, USA). FITC Annexin V Apoptosis Detection Kit I was purchased from B.D. Biosciences (Franklin Lakes, NJ, USA).

#### 4.2.2. Cell Culture

Cancer cell lines: BxPC-3 (pancreas adenocarcinoma, ATCC^®^ CRL-1687^TM^), and HT-29 (colorectal adenocarcinoma, ATCC^®^ HTB-38^TM^) and normal cell line—WI-38 (human lung fibroblasts, ATCC^®^ CCL-75TM) were obtained from American Type Culture Collection (ATCC, Rockville, MD, USA). BxPC-3 cells were grown in RPMI-1640 medium supplemented with 10% (*v*/*v*) FBS and 1% (*v*/*v*) of both antibiotics (streptomycin and penicillin). For the HT-29 cell line, RPMI-1640 medium supplemented with 10% (*v*/*v*) FBS and 1% (*v*/*v*) of both antibiotics and 1% MEM nonessential amino acids was used to ensure proper cell growth. WI-38 cells were grown in MEM medium supplemented with 10% (*v*/*v*) FBS, L-Glutamine, 25 mM Hepes, and 1% penicillin–streptomycin. MycoBlue^TM^ Mycoplasma Detector kit (Vazyme Biotech, Nanjing, China) was used at least every month for the control of mycoplasma contamination in the cell cultures.

Cells were grown at 37 °C in a humidified atmosphere of 5% CO_2_ in the air. The culture medium was changed every 24–48 h. Subculture was performed using 0.25% trypsin/EDTA after cells reached confluence.

#### 4.2.3. MTT Assay

The MTT assay, also known as the 3-(4,5-dimethylthiazol-2-yl)-2,5-diphenyltetrazolium bromide assay, is a colorimetric assay used in biological and biomedical research to determine cell viability and proliferation. The MTT assay works on the assumption that live cells with active metabolism convert MTT into formazan crystals. These crystals are insoluble; however, they can be dissolved in an organic solvent such as DMSO to produce a colorful solution. The color intensity is proportional to the number of viable cells present in the culture.

BxPC-3, HT-29, and WI-38 cells were seeded on 96-well plates at a density of approximately 8–10 × 10^3^ cells per 100 µL medium/well. Cells were allowed to grow for 24 h in controlled conditions (37 °C; 5% CO_2_). Afterward, the cells were treated with **MMZ** compounds and 5-Fluorouracil (dissolved in sterile distilled water) in concentrations ranging from 5 to 400 µM (final concentration of DMSO was < 0.5% *v/v*) or IC_50_ concentrations determined for cancer cell lines for respective time periods (24 or 72 h) and respective cancer cell lines for cell viability testing in WI-38 cells [54] in the culture medium for another 24 or 72 h. The experimental design included nontreated controls and blanks (wells without cells). Following 24 or 72 h of culture with the investigated compounds, 20 μL of MTT tetrazolium salt (5 mg/mL in PBS) was added to each well, and the plates were incubated for 3 h in a humidified environment (37 °C; 5% CO_2_). The solutions were withdrawn after incubation, and 100 μL of DMSO was added to dissolve the formazan complexes. Afterward, a spectrophotometer (microplate reader Power Wave XS BioTek Instruments, Inc., Winooski, VT, USA) reading at 570 nm was conducted. The experiments were carried out in duplicates.

GraphPad Prism 7 software was used to calculate the concentration of an **MMZ** compound reflecting a 50% growth inhibition (IC_50_). IC_50_ is defined as a concentration of tested compound that leads to a reduction in cell pool viability by 50% compared to the negative control (accepted as 100%).

$$\%\;\mathrm{cell}\;\mathrm{viability}=\frac{\left(\mathrm{Absorbance}\;\mathrm{value}\;\mathrm{of}\;\mathrm{treated}\;\mathrm{cells}-\mathrm{Absorbance}\;\mathrm{value}\;\mathrm{of}\;\mathrm{blank}\right)}{\left(\mathrm{Absorbance}\;\mathrm{value}\;\mathrm{of}\;\mathrm{untreated}\;\mathrm{cells}-\mathrm{Absorbance}\;\mathrm{value}\;\mathrm{of}\;\mathrm{blank}\right)}\times 100\%$$

#### 4.2.4. Dual Acridine Orange/Ethidium Bromide (AO/EB) Fluorescent Staining

BxPC-3 and HT-29 cells were plated at a density of 5 × 10^4^ cells per well in 12-well plates. After 24 h, the cells were treated with **MMZ-45AA** and **MMZ-140C** at concentrations corresponding to their IC_50_ values obtained from the MTT assay. The cells were exposed to the compounds for 72 h. Following the incubation period, the cells were treated with a mixture of fluorochromes (100 µM AO/EB in a 1:1 ratio, *v/v*) for 5 min at 37 °C in the absence of light.

The staining technique allowed for the differentiation of viable, apoptotic, and necrotic cells based on their varying uptake of fluorescent DNA-binding dyes and the degree of chromatin condensation in the stained nuclei. A fluorescence microscope (Olympus BX60 F5, Olympus Optical Co., Ltd., Nagano, Japan) with an excitation wavelength of 360 nm was used to analyze the cells. The results were obtained from three independent experiments.

#### 4.2.5. Annexin V-FITC and Propidium Iodide Flow Cytometry Analysis

The Annexin V-FITC Apoptosis Detection Kit was utilized to assess the induction of apoptosis following 72 h incubation with the tested **MMZ** compounds. BxPC-3 and HT-29 cells were plated at a density of 4 × 10^5^ cells in 6-well plates. After 24 h, the cells were exposed to IC_50_ concentrations of **MMZ-45AA** and **MM1-40C**, determined in the MTT assay. The experimental setup included a vehicle control with a final solvent concentration of less than 0.5% *v/v* DMSO, and cells treated with 2 µM SN-38 (the active metabolite of irinotecan) as a positive control. The cells were then incubated for an additional 72 h at 37 °C with 5% CO2.

Following the exposure period, the cells were trypsinized, transferred to cytometric tubes, and allowed to incubate for 40 min. Subsequently, they were centrifuged at 1400 rpm for 10 min at 4 °C. The supernatant was then removed, and the cell pellet was diluted in 1 mL PBS. The remaining steps were carried out according to the instructions provided by the manufacturer of the Annexin V-FITC Apoptosis Detection Kit. The results reported in this study were obtained from three independent experiments

### 4.3. Computational Analysis

#### 4.3.1. Drug-Likeness and ADMET

The Swiss-ADME web tool (http://www.swissadme.ch) [34], pkCSM (https://biosig.lab.uq.edu.au/pkcsm/) [33], PreADMET (https://preadmet.webservice.bmdrc.org/), and Protox-II (http://tox.charite.de/protox_II) [44,45] online servers (accessed on 26 February 2023) were used to estimate critical physicochemical, pharmacological, toxicological, and drug-likeness properties of tested **MMZ** compounds.

#### 4.3.2. DFT Calculations

DFT calculations were performed using Jaguar v11.5 module in Schrödinger to predict the chemical reactivity of the molecules. Hybrid DFT with Berke’s three-parameter exchange potential and Lee–Yang–Parr correlation functional (B3LYP), using basis set 6-31G++** level was used to optimize the structures. All DFT calculations were performed in an aqueous environment using PBF. Calculations such as HOMO, LUMO, and MESP were performed.

#### 4.3.3. Molecular Target Prediction

SwissTargetPrediction web server (http://www.swisstargetprediction.ch/) [50,51] and SuperPred 3.0 webserver (https://prediction.charite.de/subpages/target_prediction.php) [55] were used to fetch possible molecular targets for **MMZ** compounds in the molecular docking studies that followed. The databases were accessed on 26 February 2023.

#### 4.3.4. Molecular Docking

Molecular docking analysis was performed in an attempt to explore the potential inhibitory effects of compounds **MMZ-33**, **MMZ-39**, **MMZ-45**, **MMZ-140**, **MMZ-147**, and **MMZ-167** on the selected anticancer drug targets. **MMZ** compounds were docked against the anticancer drug targets predicted by the SwissTargetPrediction and SuperPred 3 webservers. The proposed ligands were sketched in ChemDraw 12.0 software and their three-dimensional structure was obtained following energy minimization using an MM2 force field. To obtain the ligands ready for molecular docking simulation, we used AutoDock Tools (The Scripps Research Institute, La Jolla, CA, USA) to find aromatic carbons and rotatable bonds, configure the automated torsion number, margin nonpolar hydrogens, and add Gasteiger charges.

The PDB codes for the downloaded proteins were obtained from PDB https://www.rcsb.org/ (accessed on 26 February 2023). Using AutoDockTools, the macromolecules were assigned autodock atom type (AD4), and the Gasteiger charge was added and distributed along the macromolecule. The structures were saved in PDBQT file format.

The grid box values were adjusted following the docking of reference ligands already complexed in the PDB structure after thorough observation of the drug’s conformational poses. The grid parameters for each enzyme were saved in a grid parameter file (GPF). The Autogrid utility from the Autodock suite was used to create the additional map files required for running the molecular docking simulations (Table 6).

#### 4.3.5. Molecular Dynamics

MD simulation is a complex structural analysis that provides copious dynamical structural evidence of biomacromolecules. It reveals the affluence of valuable information related to the thermodynamic stability of protein and ligand complex with time. Dynamic structural behavior of the macromolecule is imperative to expose the structure–function relationship of the macromolecular target. The observed protein–ligand interactions during the MD simulation play a vital role in the drug design and discovery process. Thus, MD simulations have been an essential and widely used method imparting successful implementation of each step involved in modern drug discovery.

Based upon the observed docking results, pharmacokinetic and toxicity profiling of the **MMZ** compounds, the macromolecular complex of compound **MMZ-45** against nuclear factor-kappa B1 (NF-KB1), ADORA1, CDK1, CDK2, polo-like kinase 1 (PLK1), TRIM24, and casein kinase 2 (CK2) were shortlisted to execute MD simulation analysis. A total of ten MD simulations were performed for the shortlisted macromolecular complexes by using Schrodinger’s Desmond module with GPU acceleration. The macromolecular complex was prepared and parameterized by using the OPLS45 force field. The generated system was neutralized and solvated by adding sodium and chloride ions and suspending it in an orthorhombic box of TIP3P water molecules so that all atoms were within 8 Å of the box edges. A 2000-step partial minimization was performed, while applying a 1000-step restraint potential of 500 Kcal/mol, using the steepest descent method followed by 1000 steps of conjugate gradients. Furthermore, 1000 steps of full minimization were executed using a conjugate gradient algorithm without restraint. A heating MD simulation from 0 to 300 K was gradually carried out by maintaining a fixed number of atoms and volume.

#### 4.3.6. Prime MM-GBSA Calculations

The Prime MM-GBSA module of Schrödinger is used to evaluate the binding free energies for the protein-ligand complexes. The protein–ligand complexes are minimized using an optimized potential liquid solvation–all atom (OPLS-AA) force field and generalized Born/Surface (GB/SA) continuum solvent model. The free energy of binding for the protein–ligand complexes is estimated using the following formula.

ΔGbind = Gcomplex − (Gprotein + Gligand)

G = EMM + GSGB + GNP

The energies of the complex, protein, and unbound ligand are represented as G_complex_, G_protein_, and G_ligand_, respectively. The molecular mechanic’s energies (EMM), in addition to SGB polar solvation model (GSGB), and nonpolar solvation (GNP) are together represented as G.

## Figures and Tables

**Table 1:** Molecular properties describing Lipinski’s rule of five predicted with SwissADME webserver (http://www.swissadme.ch/, accessed on 26 February 2023).

Compound	Molecular Weight	Hydrogen Bond Acceptors	Hydrogen Bond Donors	Consensus Log *p* Value	Drug-Likeness
**MMZ-33**	363.45	4	2	4.32	Yes; 0 violation
**MMZ-39**	397.89	4	2	4.86	Yes; 0 violation
**MMZ-45**	411.49	4	2	4.86	Yes; 1 violation: MLOGP > 4.15
**MMZ-140**	361.43	4	1	3.79	Yes; 0 violation
**MMZ-147**	393.43	6	2	3.37	Yes; 0 violation
**MMZ-167**	377.48	4	2	4.62	Yes; 0 violation

**Table 2:** Predicted ADMET properties of **MMZ** compounds.

Compound/Property	Gastrointestinal (GI) Absorption (SwissADME)	CYP2D6 Inhibitor (SwissADME/pkCSM)	CYP3A4 Inhibitor (SwissADME/pkCSM)	Blood–Brain Barrier (BBB) Permeability (SwissADME)	P-glycoprotein Substrate (SwissADME)	Ames Toxicity (pkCSM/PreADMET)	Cardiotoxicity (hERG Inhibition) (PreADMET)	Hepatotoxicity (pkCSM)
**MMZ-33**	High	Yes/No	No/No	Yes	Yes	No/No	Ambiguous	Yes
**MMZ-39**	High	Yes/No	Yes/No	Yes	Yes	Yes/No	Medium risk	Yes
**MMZ-45**	High	Yes	Yes	Yes	Yes	Yes/Yes	Ambiguous	Yes
**MMZ-140**	High	Yes	No/Yes	Yes	No	Yes/Yes	Low risk	Yes
**MMZ-147**	High	Yes/No	Yes	No	No	Yes/No	Ambiguous	Yes
**MMZ-167**	High	Yes	Yes	Yes	Yes	Yes/No	Ambiguous	Yes

**Table 3:** Toxicity profiles of **MMZ** compounds estimated using Protox-II (http://tox.charite.de/protox_II, accessed on 26 February 2023).

Compound /Property	Hepatotoxicity	Immunotoxicity	Mutagenicity	ATAD5	HSE	MMP	nrf2/ARE	TP53
**MMZ-33**	Inactive (*p* = 0.55)	Inactive (*p* = 0.98)	Inactive (*p* = 0.56)	Inactive (*p* = 0.85)	Inactive (*p* = 0.92)	Active (*p* = 0.59)	Inactive (*p* = 0.92)	Inactive (*p* = 0.8)
**MMZ-39**	Active (*p* = 0.5)	Inactive (*p* = 0.93)	Inactive (*p* = 0.64)	Inactive (*p* = 0.88)	Inactive (*p* = 0.86)	Active (*p* = 0.6)	Inactive (*p* = 0.86)	Inactive (*p* = 0.72)
**MMZ-45**	Inactive (*p* = 0.64)	Inactive (*p* = 0.99)	Inactive (*p* = 0.54)	Inactive (*p* = 0.88)	Inactive (*p* = 0.93)	Inactive (*p* = 0.61)	Inactive (*p* = 0.93)	Inactive (*p* = 0.85)
**MMZ-140**	Inactive (*p* = 0.82)	Inactive (*p* = 0.86)	Inactive (*p* = 0.51)	Inactive (*p* = 0.95)	Inactive (*p* = 0.95)	Inactive (*p* = 0.69)	Inactive (*p* = 0.95)	Inactive (*p* = 0.92)
MMZ-147	Inactive (*p* = 0.65)	Inactive (*p* = 0.99)	Inactive (*p* = 0.5)	Inactive (*p* = 0.86)	Inactive (*p* = 0.94)	Inactive (*p* = 0.56)	Inactive (*p* = 0.94)	Inactive (*p* = 0.74)
**MMZ-167**	Inactive (*p* = 0.62)	Inactive (*p* = 0.86)	Inactive (*p* = 0.6)	Inactive (*p* = 0.86)	Inactive (*p* = 0.63)	Inactive (*p* = 0.61)	Inactive (*p* = 0.93)	Inactive (*p* = 0.83)

*p*—probability.

**Table 4:** Density functional theory calculation for the **MMZ** compounds.

Compound ID	HOMO (eV)	LUMO (eV)	HLG (eV)
**MMZ-33**	−0.213	−0.058	0.15
**MMZ-39**	−0.214	−0.058	0.15
**MMZ-45**	−0.201	−0.046	0.15
**MMZ-140**	−0.201	−0.046	0.15
**MMZ-147**	−0.201	−0.045	0.15
**MMZ-148**	−0.201	−0.046	0.15
**MMZ-167**	−0.203	−0.047	0.15

**Table 5:** Binding free energy calculation for the protein–ligand complexes of the **MMZ-45** compound.

Protein	PDB id	ΔG_coulomb_^a^	ΔG_vdw_^b^	ΔG_covalent_^c^	ΔG_solv_^d^	ΔG_solvlipo_^e^	ΔG_bind_^f^
ADORA1	6D9H	−12.86	−52.55	17.9	35.45	−35.2	−51.57
CDK1	6GU6	−7.7	−47.87	15.01	38.36	−24.03	−28.53
CDK2	2FVD	−10.49	−49.28	12.96	28.05	−46.92	−66.73
CK	6TLS	−7.57	−31.17	3.54	21.06	−26.48	−41.89
NFKB1	1SVC	−16.51	−35.67	2.8	37.3	−10.43	−23.71
PLK1	3FC2	−18.94	−34.95	10.71	26.68	−22.78	−42.18
TRIM24	4YBM	−18.94	−34.95	10.71	26.68	−22.78	−42.18

^a^ contribution to the MM-GBSA free energy of binding from the Coulomb energy; ^b^ contribution to the MM-GBSA free energy of binding from the van der Waals energy; ^c^ contribution to the MM-GBSA free energy of binding from the covalent binding; ^d^ contribution to the MM-GBSA free energy of binding from the nonpolar contribution to the solvation energy due to the surface area; ^e^ contribution to the MM-GBSA free energy of binding lipophilic binding; ^f^ free energy of binding.

**Table 6:** The coordinates of the grid boxes used in the docking studies of **MMZ** compounds to currently investigated anticancer drug targets together with their PDB codes.

Drug Target	PDB Code	x-D	y-D	z-D	Spacing (Ả)	x Center	y Center	z Center
AKT1	6CCY	40	50	48	0.425	−9.801	15.312	−31.398
AURKB	4AF3	40	50	40	0.408	21.226	−21.921	−10.221
CDK1	6GU6	40	40	40	0.469	23.159	21.848	−2.268
CDK2	2FVD	40	40	40	0.397	1.231	28.133	8.792
EGFR	7VRA	40	40	40	0.397	50.153	1.467	−19.629
FGFR	5AM6	40	40	48	0.392	217.536	−7.806	24.459
FLT3	4RT7	40	50	48	0.425	−38.825	11.685	−15.423
JAK2	2B7A	40	40	40	0.419	114.221	64.945	10.271
JAK3	3PJC	40	50	40	0.403	8.857	−5.345	10.707
PLK1	3FC2	40	50	40	0.431	47.588	−5.939	9.028
PRKCQ	4Q9Z	40	50	40	0.408	21.315	−8.295	−8.006
RIPK2	5W5O	40	50	48	0.431	−1.027	16.243	94.25
ADORA1	6D9H	40	50	48	0.419	92.16	120.297	92.496
BACE1	4IVT	40	50	40	0.414	22.376	22.871	0.873
BACE2	2EWY	40	40	40	0.442	106.825	24.801	2.867
LTA4H	5N3W	40	50	48	0.431	11.962	−1.41	0.812
CAPN1	2NQG	40	40	40	0.419	13.64	8.089	−21.839
MMP16	1RM8	40	40	40	0.431	0.381	3.249	48.361
PDE2A	5U7D	40	50	48	0.408	14.119	6.752	19.46
PDE5	2H42	40	40	40	0.408	30.79	119.342	11.038
PDE4B	4KP6	40	50	40	0.386	−41.761	91.222	114.399
PDE7A	4PM0	40	50	40	0.403	−45.444	25.125	1.399
PDE10A	3WI2	40	50	40	0.414	20.523	3.048	58.144
HSD17B2	3HB4	40	42	40	0.408	11.394	6.482	−10.657
IDH1	5LGE	40	42	40	0.392	−29.141	−99.881	25.215
LDHA	6MV8	40	40	40	0.414	40.266	14.748	27.266
CYP11B1	6M7X	40	40	40	0.397	51.53	−45.46	−6.116
CYP11B2	4ZGX	40	42	40	0.419	60.088	−54.484	115.987
STAT3	6NUQ	44	60	44	0.403	13.619	54.024	−0.083
SMARCA2	5DKH	40	42	40	0.386	−12.258	38.92	7.45
UQCRB	5NMI	40	40	40	0.403	39.838	15.607	1.039
MCL1	5FDR	40	42	40	0.403	37.551	1.085	19.468
APE1	6BOW	66	66	66	0.731	9.292	−30.663	−0.237
BCHE	5LKR	40	42	40	0.408	−41.505	50.849	−12.665
C5AR	6C1R	40	40	40	0.403	12.846	1.777	−45.271
CAPN1	1ZCM	40	40	40	0.386	−21.825	5.536	34.882
CHRM4	5DSG	40	42	40	0.392	50.41	8.435	63.576
CHRM5	6OL9	40	40	40	0.431	35.173	23.793	−40.677
CHRNA4	6UR8	40	40	40	0.403	124.113	145.382	189.741
CK	6TLS	44	44	44	0.403	77.273	7.948	21.258
CLK4	6FYV	40	40	40	0.414	−28.205	22.947	−18.2
COX2	5F19	40	42	40	0.436	27.852	30.441	63.17
CPAR	6C1R	40	40	40	0.403	12.846	1.777	−45.271
CPSD	4OD9	44	44	44	0.408	−3.204	12.697	−34.841
CYP3A4	5VCC	40	40	40	0.425	−23.77	−27.5	−11.384
DUSP3	3F81	40	42	40	0.397	−0.908	0.681	−6.463
FPR2	6OMM	40	40	40	0.486	116.151	131.184	111.263
GLUT1	6THA	40	40	40	0.392	18.054	59.12	11.134
GNAI1	6N4B	40	40	40	0.397	92.223	131.93	120.478
HADH2	2O23	40	42	40	0.414	19.88	13.935	−12.26
HERG	5VA1	100	82	108	1.000	93.575	57.237	60.469
MDM4	6Q9Y	40	40	40	0.392	−5.108	10.302	−14.609
NFKB1	1SVC	48	58	58	0.394	28.358	17.443	43.771
NR1A1	3ILZ	40	42	40	0.425	28.128	38.913	32.411
NR1I2	6TFI	40	40	40	0.403	51.713	36.805	15.99
PRCP	3N2Z	80	84	96	0.686	51.603	32.164	72.575
PSMA2	6KWY	40	40	40	0.436	238.36	189.562	104.143
SLC1A3	5LM4	40	42	40	0.397	−476.806	301.433	12.118
TDP1	6N0D	40	40	40	0.392	8.387	−14.555	−34.733
TRIM24	4YBM	44	44	44	0.347	36.436	−18.263	−32.015

## Data Availability

The data presented in this study are available in the main text of this article/Supplementary Materials of this article or on request from the corresponding author.

## Supplementary Materials

The following supporting information can be downloaded at: https://www.mdpi.com/article/10.3390/molecules28207230/s1. Figure S1. ^1^H NMR spectra of (*S*,*S*) and (*R*,*R*)-methyl ((2-hydroxynaphthalen-1-yl)(phenyl)methyl)-d-valinate (**MMZ-33D**). Figure S2. ^1^H NMR spectra of (*S*,*S*) and (*R*,*R*)-methyl ((4-chlorophenyl)(2-hydroxynaphthalen-1-yl)methyl)-d-valinate (**MMZ-39AA**). Figure S4. ^1^H and ^13^C NMR spectra of (*S*,*R*) and (*R*,*S*)-methyl ((2-hydroxynaphthalen-1-yl)(phenyl)methyl)-l-phenylalaninate (**MMZ-45B**). Figure S5. ^1^H and ^13^C NMR spectra of (*S*,*S*) and (*R*,*R*)-methyl ((2-hydroxynaphthalen-1-yl)(phenyl)methyl)-l-prolinate (**MMZ-140C**). Figure S6. ^1^H and ^13^C NMR spectra of (*S*,*S*) and (*R*,*R*)-dimethyl ((2-hydroxynaphthalen-1-yl)(phenyl)methyl)-l-aspartate (**MMZ-147B**). Figure S7. ^1^H and ^13^C NMR spectra of (*S*,*R*) and (*R*,*S*)-dimethyl ((2-hydroxynaphthalen-1-yl)(phenyl)methyl)-L-aspartate (**MMZ-147C**). Figure S8. ^1^H and ^13^C NMR spectra of (*S*,*S*) and (*R*,*R*)-dimethyl ((2-hydroxynaphthalen-1-yl)(phenyl)methyl)-l-glutamate (**MMZ-148B**). Figure S9. ^1^H and ^13^C NMR spectra of (*S*,*R*) and (*R*,*S*)-dimethyl ((2-hydroxynaphthalen-1-yl)(phenyl)methyl)-l-glutamate (**MMZ-148C**). Figure S10. ^1^H and ^13^C NMR spectra of (*S*,*S*) and (*R*,*R*)-methyl ((2-hydroxynaphthalen-1-yl)(phenyl)methyl)-l-leucinate (**MMZ-167C**). Figure S11. HOMO and LUMO distribution profile of the compounds. (A) **MMZ-33**, (B) **MMZ-39**, (C) **MMZ-45**, (D) **MMZ-140**, (E) **MMZ-147**, (F) **MMZ-148**, (G) **MMZ-167**. Table S1. Results of 24-h and 72-h MTT assay. IC_50_ values from two independent experiments (IC_50(1)_ and IC_50(2)_) with corresponding coefficients of determination (R^2^), 95% confidence interval (95%CI) values and calculated mean IC_50_ values ± SD. Table S2. The predicted molecular targets **MMZ** compounds using SuperPred 3.0 webserver. Table S3. Molecular docking results of Betti reaction products (**MMZ**-compounds) as well as all the reference ligands.

## Sample Availability

Not available.

## Abbreviations

ADMET	absorption, distribution, metabolism, excretion, and toxicity
ADORA1	adenosine A1 receptor
AKT	protein kinase B
AO	acridine orange
ARE	antioxidant response element
ATAD5	ATPase family AAA domain-containing protein 5
BBB	blood–brain barrier
CADD	computer-assisted drug design
CDK2	cyclin-dependent kinase 2
CYP	cytochrome P
DFT	density functional theory
DMSO	dimethyl sulfoxide
EB	ethidium bromide
EMM	molecular mechanic energies
ERK	extracellular signal-regulated kinase
FBS	fetal bovine serum
FITC	fluorescein isothiocyanate
GB/SA	generalized Born/Surface
GI	gastrointestinal
GNP	nonpolar solvation
GPF	grid parameter file
GSGB	SGB polar solvation model
GSK-3β	glycogen synthase kinase-3 beta
Herg	ether-a-go-go-related gene
HLG	HOMO-LUMO energy gap
HOMO	highest occupied molecular orbital
HSE	heat shock response
JNK	c-Jun N-terminal kinase
LQTS	long QT syndrome
LUMO	lowest occupied molecular orbital
MCR	multicomponent reactions
MDR	multidrug resistance
MESP	molecular electrostatic potential
MMP	mitochondrial membrane potential
NF-KB1	nuclear factor-kappa B1
OPLS-AA	optimized potential liquid solvation-all atom
PBS	buffered saline
PDB	protein data bank
P-gp	P-glycoprotein
PI	propidium iodide
PI3K	phosphatidylinositol 3-kinase
PLK1	polo-like kinase 1
RMSD	root-mean-square deviation
RMSF	root-mean-square fluctuation
SLC6A14	sodium- and chloride-dependent neutral and basic amino acid transporter B(0+)
TP53	cellular tumor antigen p53
TRIM24	transcription intermediary factor 1-alpha
