# Supplementary material for: Synthesis, Computational, and Anticancer In Vitro Investigations of Aminobenzylnaphthols Derived from 2-Naphtol, Benzaldehydes, and α-Aminoacids via the Betti Reaction
Source: Molecules. 2023 Oct 23;28(20):7230. doi: 10.3390/molecules28207230 (PMC10609152; doi:10.3390/molecules28207230)
Supplement: Supplementary file 1 [file molecules-28-07230-s001.zip › molecules-2519795-supplementary.pdf]

Supplementary Information

Synthesis, Computational, and Anticancer In Vitro Investigations of Aminobenzylnaphthols Derived from 2-Naphtol, Benzaldehydes, and α-Aminoacids via the Betti Reaction

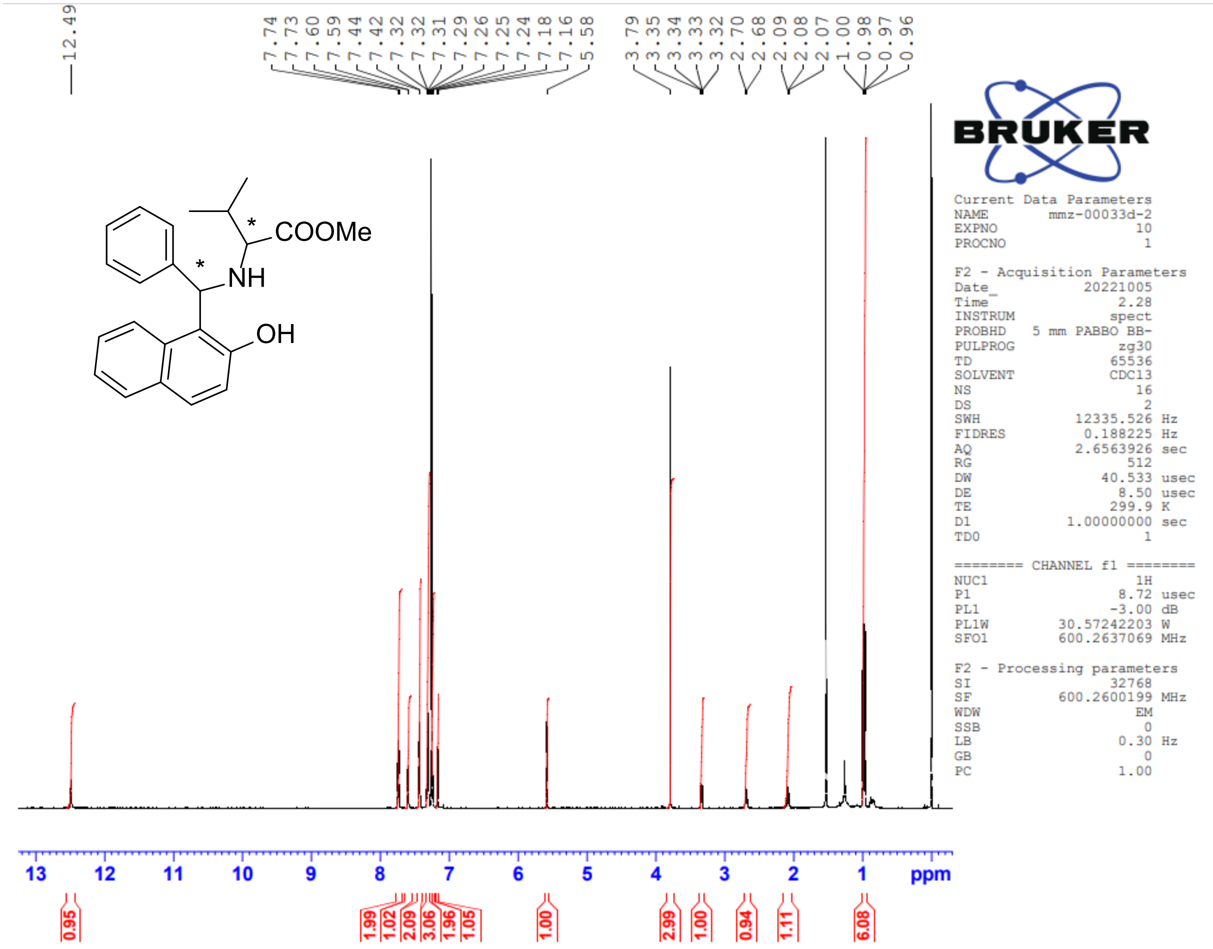

Figure S1. ^{1}H NMR spectra of (*S*,*S*) and (*R*,*R*)-methyl ((2-hydroxynaphthalen-1-yl)(phenyl)methyl)-D-valinate (**MMZ-33D**).

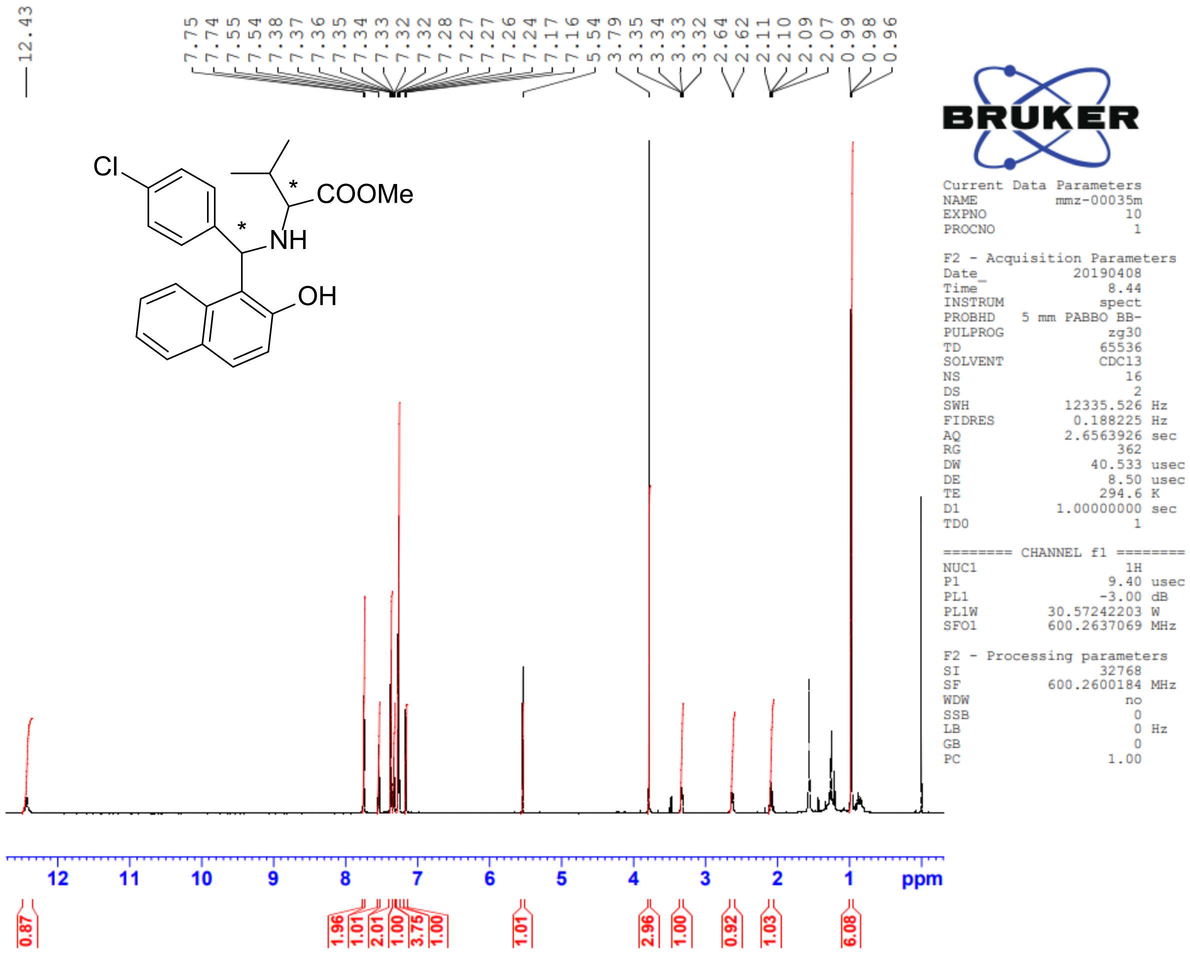

Figure S2. ^{1}H NMR spectra of (*S*,*S*) and (*R*,*R*)-methyl ((4-chlorophenyl)(2-hydroxynaphthalen-1-yl)methyl)-D-valinate (**MMZ-39AA**).

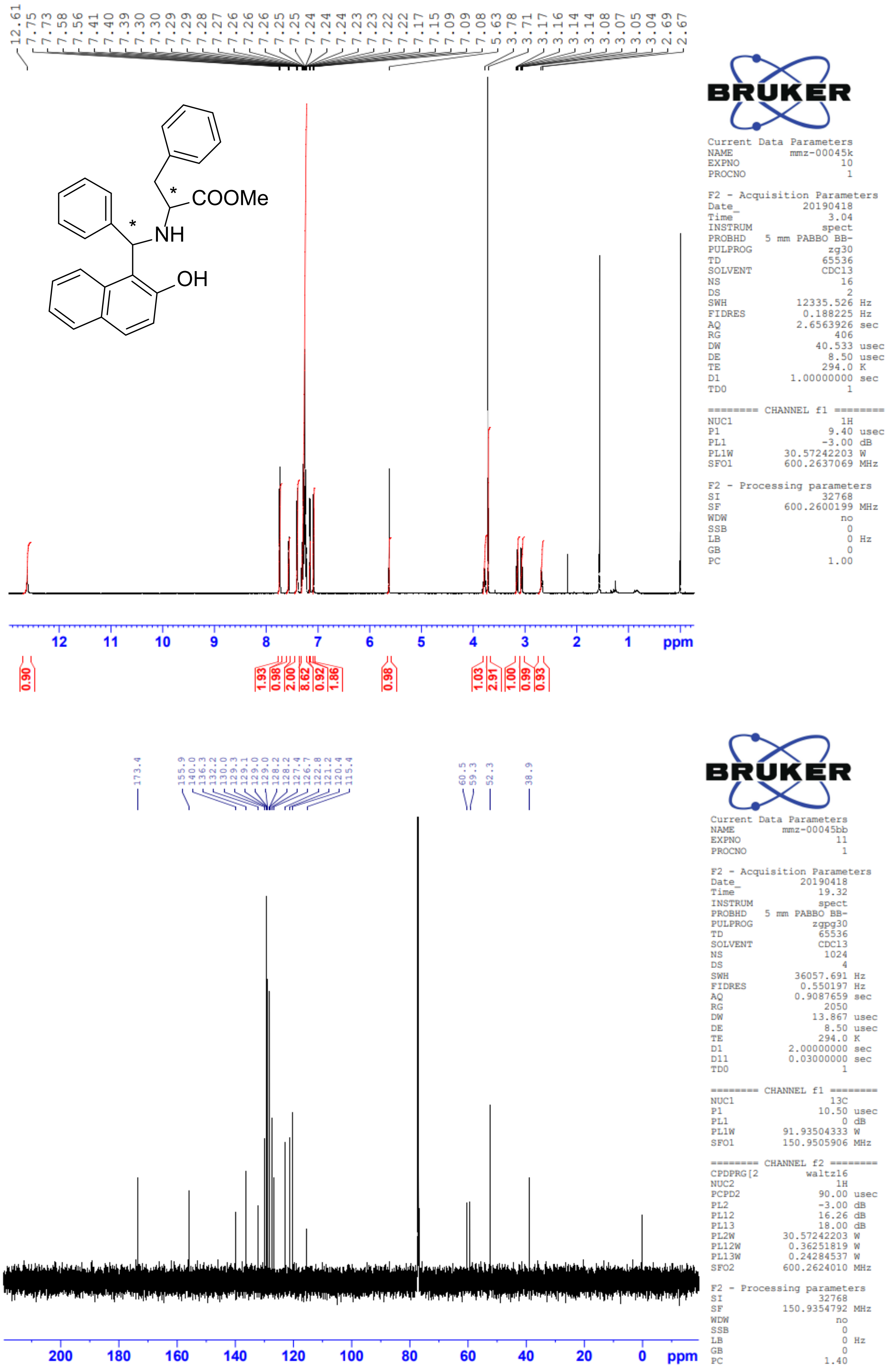

Figure S3. ^{1}H and ^{13}C NMR spectra of (*S*,*S*) and (*R*,*R*)-methyl ((2-hydroxynaphthalen-1-yl)(phenyl)methyl)-L-phenylalaninate (**MMZ-45AA**).

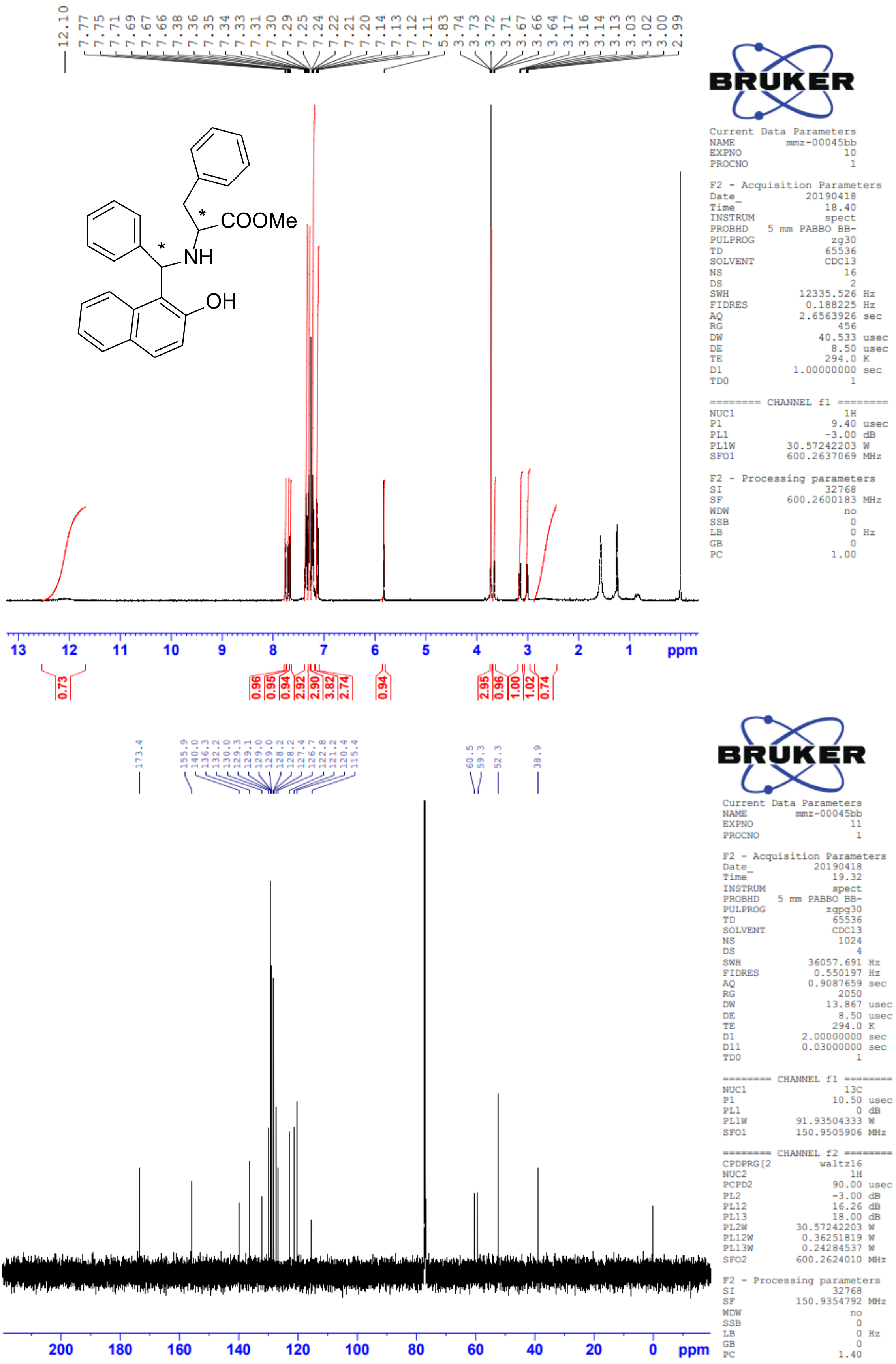

Figure S4. ^{1}H and ^{13}C NMR spectra of (*S*,*R*) and (*R*,*S*)-methyl ((2-hydroxynaphthalen-1-yl)(phenyl)methyl)-L-phenylalaninate (**MMZ-45B**).

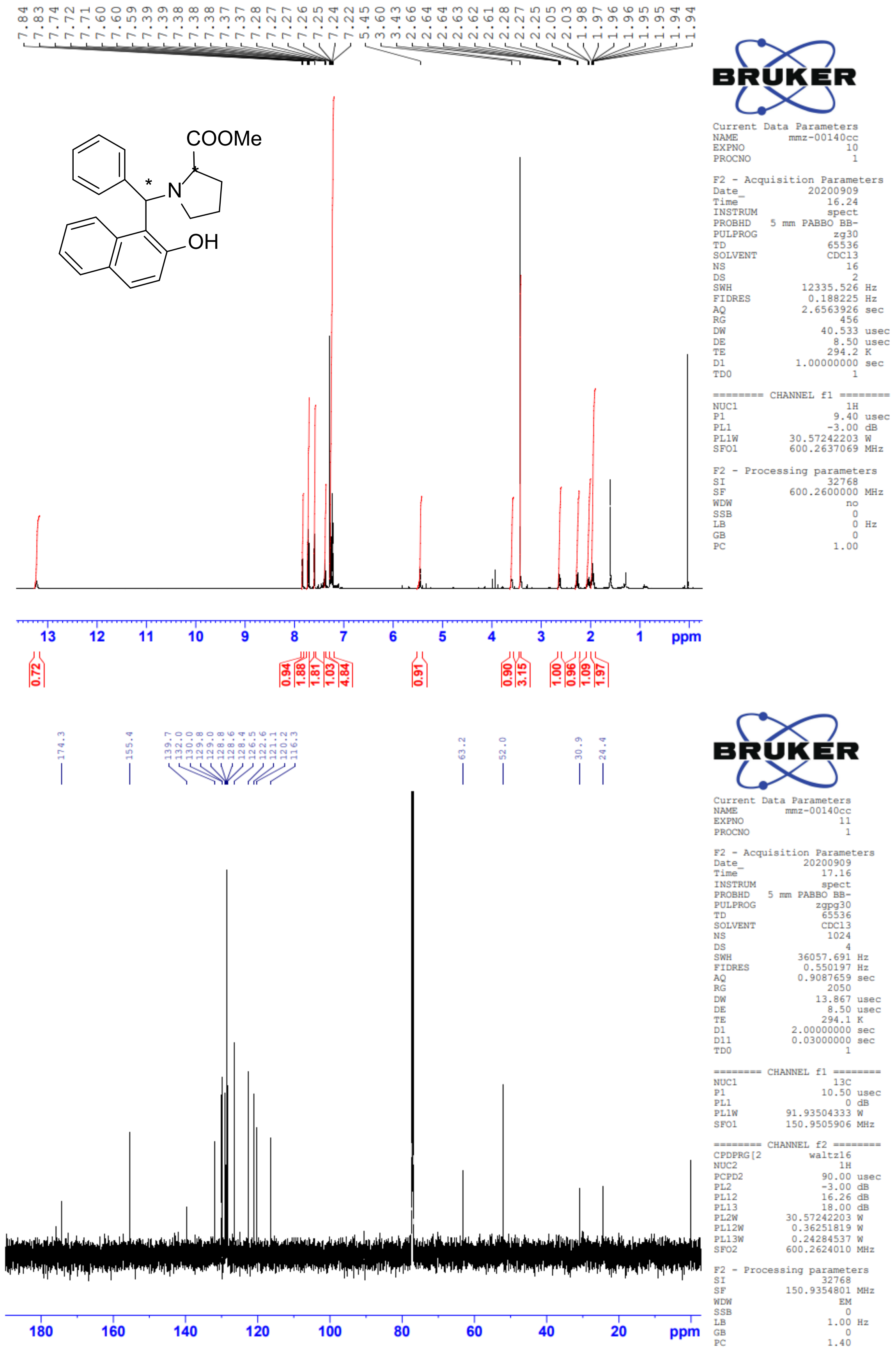

Figure S5. ^{1}H and ^{13}C NMR spectra of (*S*,*S*) and (*R*,*R*)-methyl ((2-hydroxynaphthalen-1-yl)(phenyl)methyl)-L-prolinate (**MMZ-140C**).

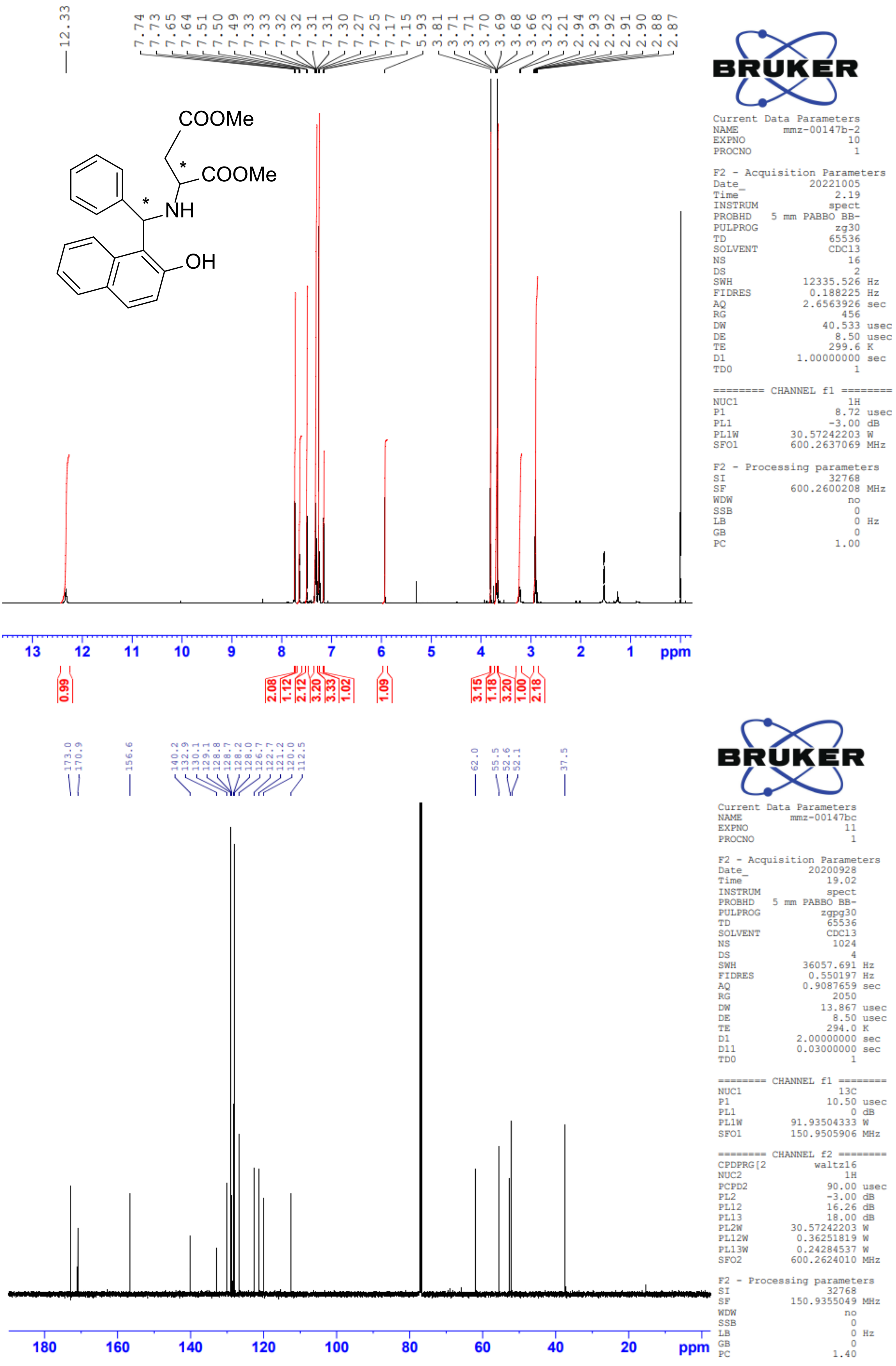

Figure S6. ^{1}H and ^{13}C NMR spectra of (*S*,*S*) and (*R*,*R*)-dimethyl ((2-hydroxynaphthalen-1-yl)(phenyl)methyl)-L-aspartate (**MMZ-147B**).

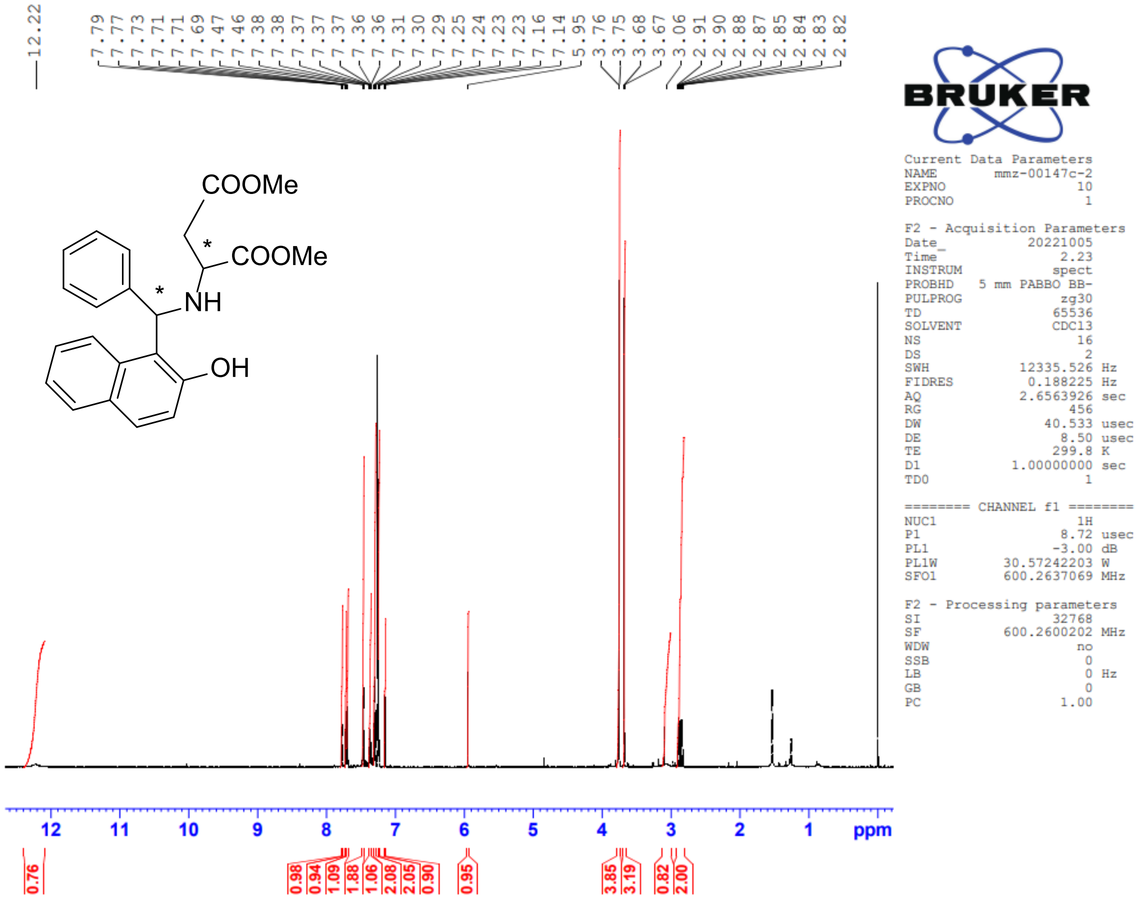

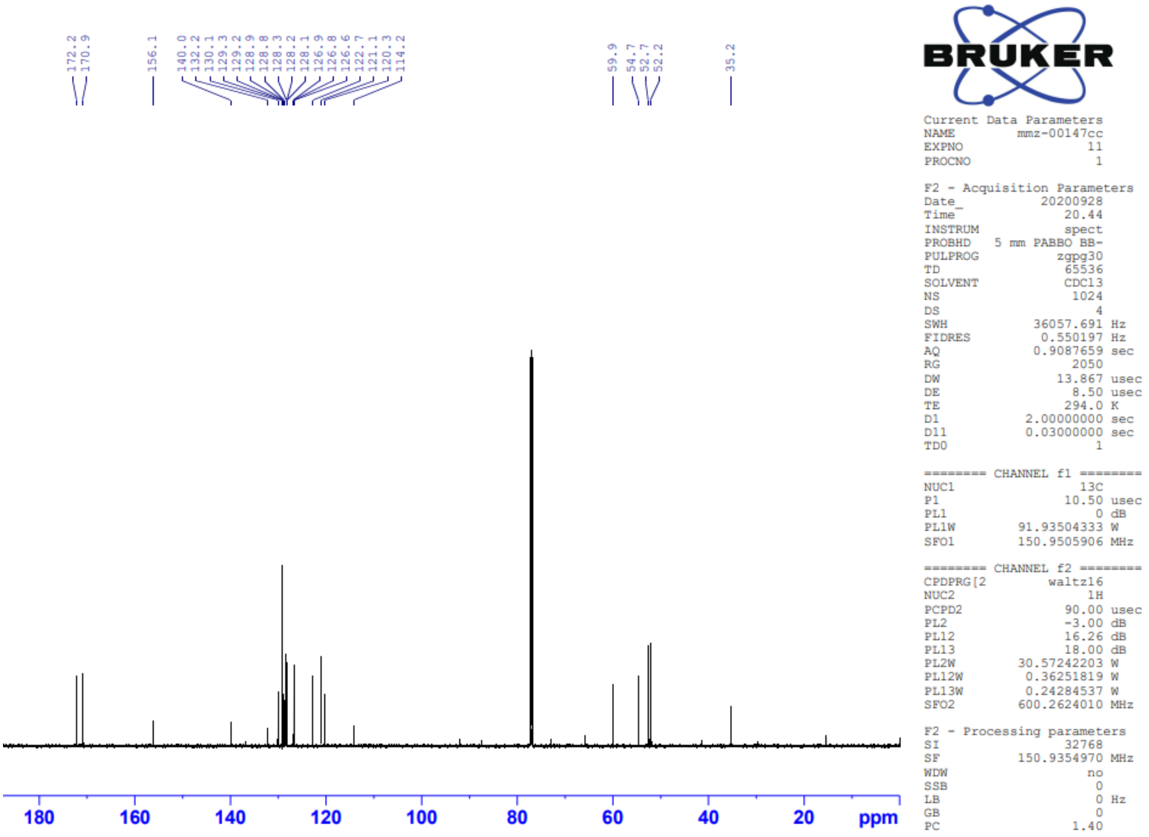

Figure S7. ^{1}H and ^{13}C NMR spectra of (*S*,*R*) and (*R*,*S*)-dimethyl ((2-hydroxynaphthalen-1-yl)(phenyl)methyl)-L-aspartate (**MMZ-147C**).

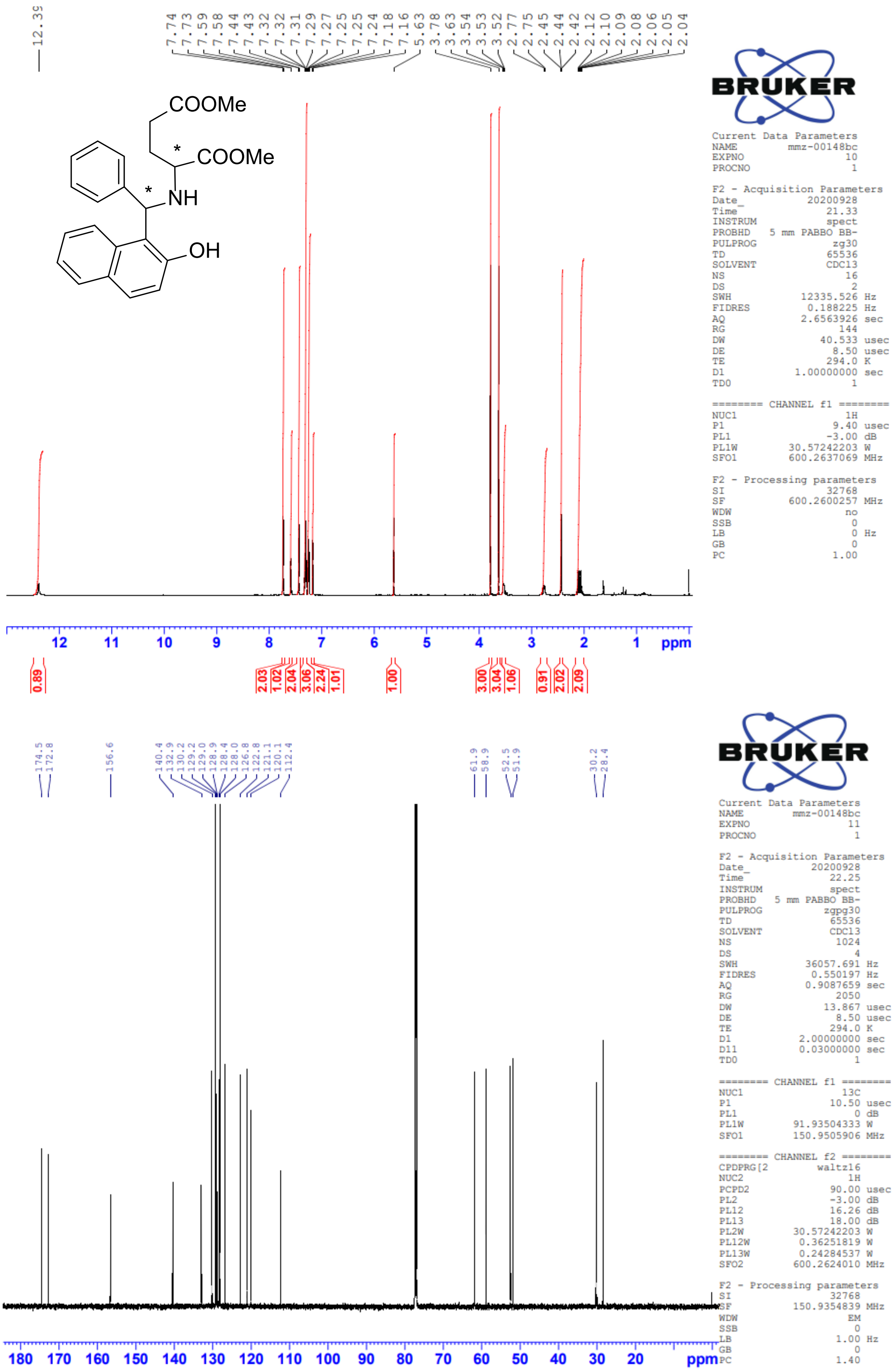

Figure S8. ^{1}H and ^{13}C NMR spectra of (*S*,*S*) and (*R*,*R*)-dimethyl ((2-hydroxynaphthalen-1-yl)(phenyl)methyl)-L-glutamate (**MMZ-148B**).

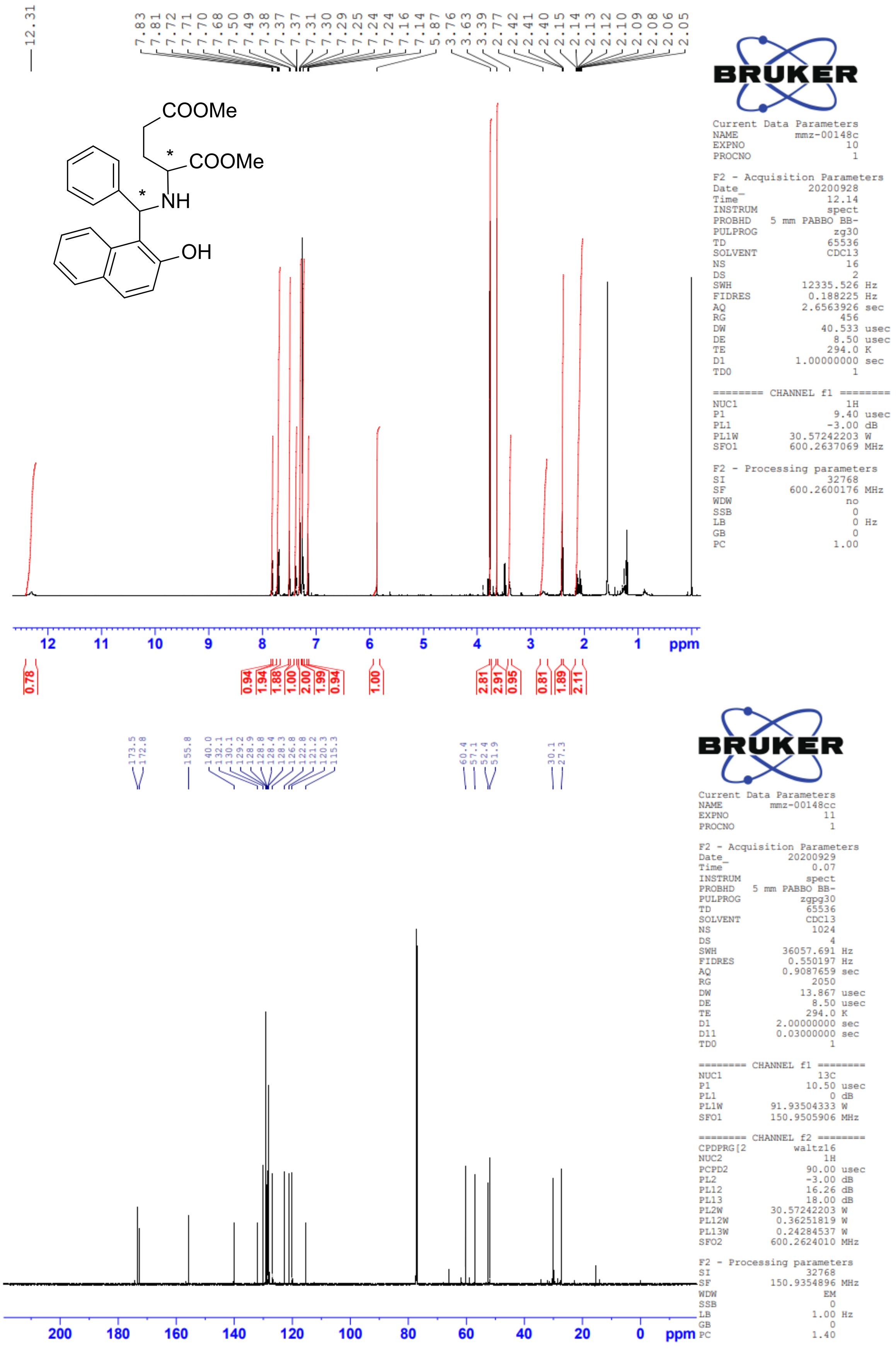

Figure S9. ^{1}H and ^{13}C NMR spectra of (*S*,*R*) and (*R*,*S*)-dimethyl ((2-hydroxynaphthalen-1-yl)(phenyl)methyl)-L-glutamate (**MMZ-148C**).

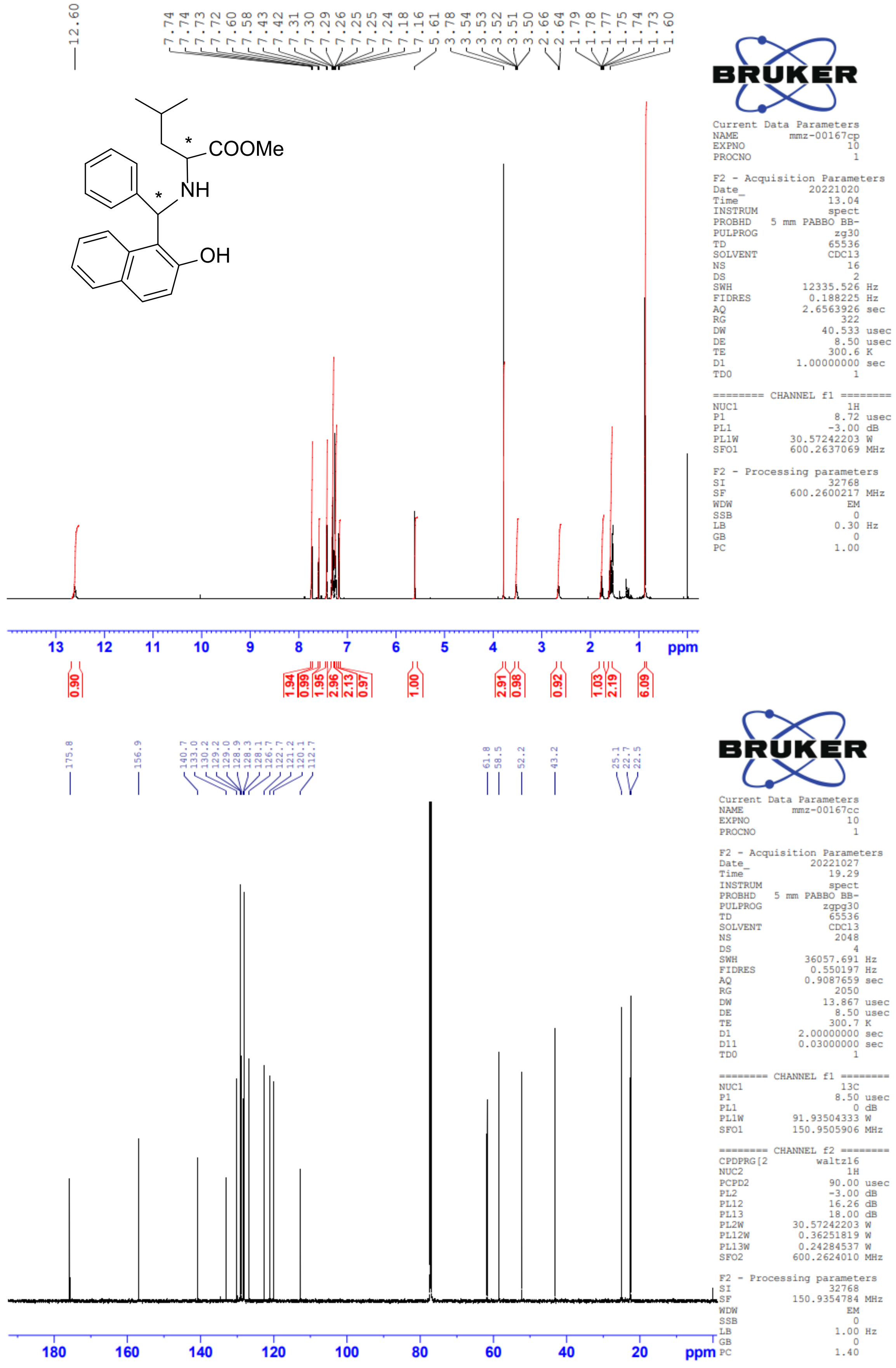

Figure S10. ^{1}H and ^{13}C NMR spectra of (*S*,*S*) and (*R*,*R*)-methyl ((2-hydroxynaphthalen-1-yl)(phenyl)methyl)-L-leucinate (**MMZ-167C**).

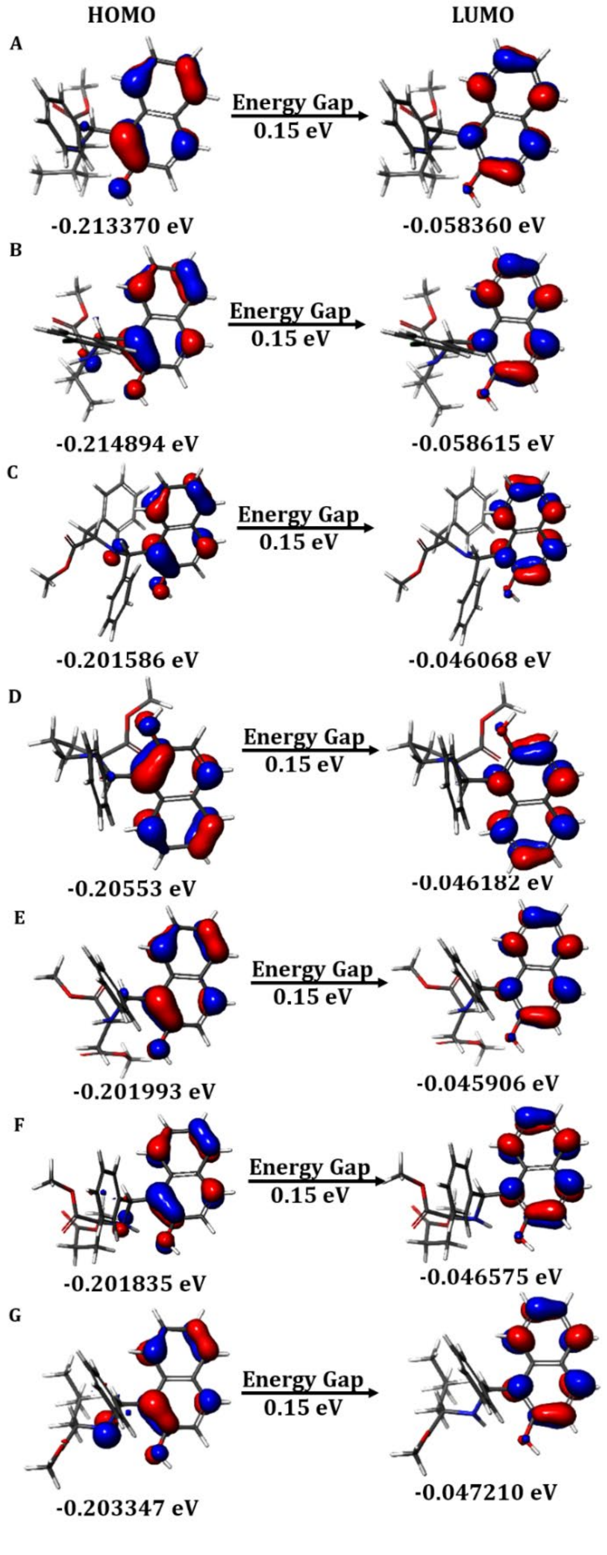

Figure S11. HOMO and LUMO distribution profile of the compounds. **A) MMZ-33**, **B) MMZ-39**, **C) MMZ-45**, **D) MMZ-140**, **E) MMZ-147**, **F) MMZ-148**, **G) MMZ-167**

Table S1. Results of 24-h and 72-h MTT assay. IC_{50} values from two independent experiments ($IC_{50(1)}$ and $IC_{50(2)}$) with corresponding coefficients of determination (R^2), 95% confidence interval (95%CI) values and calculated mean IC_{50} values ± SD.

MMZ compound	$IC_{50(1)}$	R^2_1	95%CI of IC_{50}	$IC_{50(2)}$	R^2_2	95%CI of IC_{50}	Mean IC_{50} ± SD
			BxPC-3 cell line				
			24-h				
MMZ-33D	47.82	0.99	45.01-50.90	50.78	0.92	39.76-55.82	49.3 ± 2.09
MMZ-39AA	61.40	0.97	55.96-67.69	60.26	0.98	55.74-60.69	60.83 ± 0.8
MMZ-45AA	58.07	0.92	44.26-76.46	37.97	0.98	33.92-47.09	48.02 ± 14.2
MMZ-45B	58.51	0.92	43.78-77.85	40.5	0.95	39.87-42.8	49.28 ± 13
MMZ-140C	23.51	0.97	19.46-27.49	36.8	0.98	34.02-39.09	30.15 ± 9.39
MMZ-147B	44.77	0.97	38.24-52.32	59.7	0.98	42.08-61,24	52.23 ± 10,5
MMZ-147CE	54.06	0.95	43.35-67.25	40.96	0.99	38,08-48,08	47.51 ± 9.26
MMZ-167C	71.40	0.93	55.94-92.40	60.98	0.97	58.83-92.09	66.19 ± 7.36
5-Fluorouracil (reference drug)	49.36	0.85	31.35-74.53	28.61	0.83	12.78-44.53	38.99 ± 14.67
			72-h				
MMZ-33D	50.02	0.95	42.83–58.17	28.7	0.95	23.72–33.96	39.36 ± 15.08
MMZ-39AA	44.78	0.91	35.25–54.11	41.59	0.86	31.37–51.07	43.19 ± 2.26
MMZ-45AA	12.1	0.87	6.052–18.54	14.4	0.86	6.83–22.1	13.26 ± 1.6
MMZ-45B	35.6	0.96	29.32–42.35	24.66	0.96	20.04–29.43	30.13 ± 7.73
MMZ-140C	31.65	0.94	24.2–39.64	33.19	0.9	23.94–43.05	32.42 ± 1.09
MMZ-147B	69.81	0.76	47.55–113.6	39.29	0.99	36.04–42.59	54.55 ± 21.58
MMZ-147CE	59.58	0.87	45.72–78.18	34.09	0.84	20.83–49.06	46.84 ± 18.02
MMZ-167C	32.87	0.95	26.48–39.36	31.24	0.96	27–35.76	32.06 ± 1.15
5-Fluorouracil (reference drug)	14.78	0.94	6.31–23.85	12.08	0.96	5.94–18.44	13.43 ± 1.9
			HT-29 cell line				
			24-h				
MMZ-33D	60.44	0.95	51.44-72.81	61.89	0.98	49.87-69.4	61.1 ± 1.02
MMZ-39AA	62.13	0.96	55.04-70.74	64.05	0.98	56.74-72.86	63.09 ± 1.36

MMZ-45AA	40.30	0.92	30.66-52.71	48.07	0.98	32.56-55,06	44.18 ± 5.49
MMZ-45B	29.00	0.96	23.54-34.65	34.56	0.97	31.02-38.9	31.78 ± 3.93
MMZ-140C	35.49	0.92	27.48-45.51	40.02	0.94	33.45-51.26	37.76 ± 3.2
MMZ-147B	44.24	0.92	34.17-56.48	46.78	0.93	37.27-59.02	45.51 ± 1.77
MMZ-147CE	113.0	0.74	73.61-168.5	110	0.85	78.02-154.2	111.5 ± 2.12
MMZ-167C	47.71	0.86	36.15-63.87	50.04	0.92	39.08-64.02	48,9 ± 1.65
5-Fluorouracil (reference drug)	48.78	0.87	28.70-74.03	55.74	0.74	23.80-103.1	52.26 ± 4.9
			72-h				
MMZ-33D	32.31	0.94	25.27–39.44	43.42	0.94	35.48–51.3	37.87 ± 7.856
MMZ-39AA	50.64	0.89	39.39–60.75	65.58	0.98	63.83–67.33	58.11 ± 10.56
MMZ-45AA	12.24	0.96	8.75–16.17	11.89	0.97	9.134–14.78	12.07 ± 0.2475
MMZ-45B	29.63	0.97	24.96–34.47	38.67	0.85	28.78–48.19	34.15 ± 6.392
MMZ-140C	9.014	0.94	4.666–13.74	14.08	0.93	8.634–19.84	11.55 ±3.582
MMZ-147B	12.19	0.97	9.162–15.34	17.56	0.61	1.06–40.32	14.88 ± 3.797
MMZ-147CE	37.12	0.89	24.78–51.84	46.06	0.94	1.584–1.738	41.59 ± 6.322
MMZ-167C	27.82	0.87	16.88–36.62	28.35	0.99	25.74–31.04	28.09 ± 0.3748
5-Fluorouracil (reference drug)	5.16	0.98	2.486–8.625	3.6	0.99	1.9–5.94	4.38 ± 1.1

Table S2. The predicted molecular targets **MMZ** compounds using SuperPred 3.0 webserver.

Target Name	ChEMBL-ID	UniProt ID	PDB Visualization	Probability	Model accuracy
			**MMZ-33**		
DNA-(apurinic or apyrimidinic site) lyase	CHEMBL5619	P27695	6BOW	97%	91%
Endoplasmic reticulum-associated amyloid beta-peptide-binding protein	CHEMBL4159	Q99714	2O23	94%	70%
Transcription intermediary factor 1-alpha	CHEMBL3108638	O15164	4YBM	93%	96%
Thyroid hormone receptor alpha	CHEMBL1860	P10827	3ILZ	93%	99%
Cyclooxygenase-1	CHEMBL221	P23219	6Y3C	90%	90%
Glucose transporter	CHEMBL2535	P11166	6THA	90%	99%
Casein kinase II alpha/beta	CHEMBL3038477	P67870	6TLS	90%	99%
Cathepsin B	CHEMBL4072	P07858	3PBH	88%	94%
Nuclear receptor ROR-beta	CHEMBL3091268	Q92753	N/A	88%	95,5%
Nuclear factor NF-kappa-B p105 subunit	CHEMBL3251	P19838	1SVC	88%	96%
Muscarinic acetylcholine receptor M4	CHEMBL1821	P08173	5DSG	87%	94%
Cathepsin D	CHEMBL2581	P07339	4OD9	84,9%	99%
Glycine transporter 2	CHEMBL3060	Q9Y345	N/A	84%	99%
Muscarinic acetylcholine receptor M5	CHEMBL2035	P08912	6OL9	83%	95%
Pregnane X receptor	CHEMBL3401	O75469	6TFI	82%	95%
Neuronal acetylcholine receptor; alpha4/beta4	CHEMBL1907591	P30926	6UR8	81%	100%
Hypoxia-inducible factor 1 alpha	CHEMBL4261	Q16665	4H6J	80,3%	85%

Nuclear factor erythroid 2-related factor 2	CHEMBL1075094	Q16236	2FLU	80%	96%
			**MMZ-39**		
DNA-(apurinic or apyrimidinic site) lyase	CHEMBL5619	P27695	6BOW	99%	91%
Endoplasmic reticulum-associated amyloid beta-peptide-binding protein	CHEMBL4159	Q99714	2O23	98%	70%
Cathepsin B	CHEMBL4072	P07858	3PBH	97%	94%
Pregnane X receptor	CHEMBL3401	O75469	6TFI	94%	95%
Transcription intermediary factor 1-alpha	CHEMBL3108638	O15164	4YBM	92%	96%
Thyroid hormone receptor alpha	CHEMBL1860	P10827	3ILZ	91%	99%
Lysosomal Pro-X carboxypeptidase	CHEMBL2335	P42785	3N2Z	91%	100%
Dipeptidyl peptidase II	CHEMBL3976	Q9UHL4	4EBB	90%	92%
Cyclooxygenase-1	CHEMBL221	P23219	6Y3C	88%	90%
C5a anaphylatoxin chemotactic receptor	CHEMBL2373	P21730	6C1R	87%	93%
Beta-glucuronidase	CHEMBL2728	P08236	3HN3	87%	78%
Excitatory amino acid transporter 1	CHEMBL3085	P43003	5LM4	87%	95%
Proteasome component C5	CHEMBL4208	P20618	6KWY	86%	90%
Nuclear receptor ROR-beta	CHEMBL3091268	Q92753	N/A	86%	95,5%
Dual specificity protein kinase CLK4	CHEMBL4203	Q9HAZ1	6FYV	85%	94%
Nuclear factor NF-kappa-B p105 subunit	CHEMBL3251	P19838	1SVC	84%	96%
Cannabinoid CB1 receptor	CHEMBL218	P21554	6N4B	83%	97%

Nuclear factor erythroid 2-related factor 2	CHEMBL1075094	Q16236	2FLU	81%	96%
Glucose transporter	CHEMBL2535	P11166	6THA	81%	99%
Dual specificity protein phosphatase 3	CHEMBL2635	P51452	3F81	81%	94%
Tyrosyl-DNA phosphodiesterase 1	CHEMBL1075138	Q9NUW8	6N0D	80%	71%
			**MMZ-45**		
Endoplasmic reticulum-associated amyloid beta-peptide-binding protein	CHEMBL4159	Q99714	2O23	99%	70%
Cyclooxygenase-1	CHEMBL221	P23219	6Y3C	99%	90%
DNA-(apurinic or apyrimidinic site) lyase	CHEMBL5619	P27695	6BOW	95%	91%
Cathepsin D	CHEMBL2581	P07339	4OD9	94,8%	99%
Protein Mdm4	CHEMBL1255126	O15151	6Q9Y	91%	90,2%
Nuclear receptor ROR-beta	CHEMBL3091268	Q92753	N/A	91%	95,5%
Nuclear factor NF-kappa-B p105 subunit	CHEMBL3251	P19838	1SVC	91%	96%
Tyrosyl-DNA phosphodiesterase 1	CHEMBL1075138	Q9NUW8	6N0D	91%	71%
Transcription intermediary factor 1-alpha	CHEMBL3108638	O15164	4YBM	91%	96%
Thyroid hormone receptor alpha	CHEMBL1860	P10827	3ILZ	90%	99%
Glycine transporter 2	CHEMBL3060	Q9Y345	N/A	90%	99%
Muscarinic acetylcholine receptor M5	CHEMBL2035	P08912	6OL9	87%	95%
Calpain 1	CHEMBL3891	P07384	1ZCM	85%	93%
Glucose transporter	CHEMBL2535	P11166	6THA	84%	99%
Kruppel-like factor 5	CHEMBL1293249	Q13887	N/A	83%	86%

Peptidyl-prolyl cis-trans isomerase NIMA-interacting 1	CHEMBL2288	Q13526	1PIN	82,4%	92%
Dipeptidyl peptidase II	CHEMBL3976	Q9UHL4	4EBB	82%	92%
G-protein coupled receptor 55	CHEMBL1075322	Q9Y2T6	N/A	82%	78%
Casein kinase II alpha/beta	CHEMBL3038477	P67870	6TLS	81%	99%
Neuronal acetylcholine receptor; alpha4/beta4	CHEMBL1907591	P30926	6UR8	81%	100%
Dual specificity protein kinase CLK4	CHEMBL4203	Q9HAZ1	6FYV	80%	94%
			**MMZ-140**		
Endoplasmic reticulum-associated amyloid beta-peptide-binding protein	CHEMBL4159	Q99714	2O23	99%	70%
DNA-(apurinic or apyrimidinic site) lyase	CHEMBL5619	P27695	6BOW	96%	91%
Butyrylcholinesterase	CHEMBL1914	P06276	5LKR	95%	95%
Transcription intermediary factor 1-alpha	CHEMBL3108638	O15164	4YBM	95%	96%
Casein kinase II alpha/beta	CHEMBL3038477	P67870	6TLS	94%	99%
Nuclear factor NF-kappa-B p105 subunit	CHEMBL3251	P19838	1SVC	93%	96%
Cytochrome P450 3A4	CHEMBL340	P08684	5VCC	92%	91%
Cyclooxygenase-1	CHEMBL221	P23219	6Y3C	90%	90%
Thyroid hormone receptor alpha	CHEMBL1860	P10827	3ILZ	89%	99%
Cathepsin D	CHEMBL2581	P07339	4OD9	89%	99%
Glucose transporter	CHEMBL2535	P11166	6THA	88%	99%
Cyclooxygenase-2	CHEMBL230	P35354	5F19	87%	90%

HERG	CHEMBL240	Q12809	5VA1	84%	90%
C5a anaphylatoxin chemotactic receptor	CHEMBL2373	P21730	6C1R	83%	93%
Hypoxia-inducible factor 1 alpha	CHEMBL4261	Q16665	4H6J	79,9%	85%
ADAM10	CHEMBL5028	O14672	6BE6	80%	97,5%
			**MMZ-147**		
Endoplasmic reticulum-associated amyloid beta-peptide-binding protein	CHEMBL4159	Q99714	2O23	99%	70%
DNA-(apurinic or apyrimidinic site) lyase	CHEMBL5619	P27695	6BOW	98%	91%
Cyclooxygenase-1	CHEMBL221	P23219	6Y3C	97%	90%
Tyrosyl-DNA phosphodiesterase 1	CHEMBL1075138	Q9NUW8	6N0D	93%	71%
Nuclear factor NF-kappa-B p105 subunit	CHEMBL3251	P19838	1SVC	92%	96%
Transcription intermediary factor 1-alpha	CHEMBL3108638	O15164	4YBM	91%	96%
Thyroid hormone receptor alpha	CHEMBL1860	P10827	3ILZ	91%	99%
Muscarinic acetylcholine receptor M5	CHEMBL2035	P08912	6OL9	89%	95%
Glycine transporter 2	CHEMBL3060	Q9Y345	N/A	88%	99%
Nuclear receptor ROR-beta	CHEMBL3091268	Q92753	N/A	87%	95,5%
Casein kinase II alpha/beta	CHEMBL3038477	P67870	6TLS	86,8%	99%
Cathepsin D	CHEMBL2581	P07339	4OD9	86%	99%
Kruppel-like factor 5	CHEMBL1293249	Q13887	N/A	85%	86%
ADAM10	CHEMBL5028	O14672	6BE6	81%	97,5%
Glucose transporter	CHEMBL2535	P11166	6THA	80%	99%

Nuclear factor erythroid 2-related factor 2	CHEMBL1075094	Q16236	2FLU	80%	96%
			**MMZ-167**		
Endoplasmic reticulum-associated amyloid beta-peptide-binding protein	CHEMBL4159	Q99714	2O23	98%	70%
Cyclooxygenase-1	CHEMBL221	P23219	6Y3C	98%	90%
DNA-(apurinic or apyrimidinic site) lyase	CHEMBL5619	P27695	6BOW	97%	91%
Nuclear factor NF-kappa-B p105 subunit	CHEMBL3251	P19838	1SVC	94%	96%
Cathepsin D	CHEMBL2581	P07339	4OD9	94,1%	99%
Thyroid hormone receptor alpha	CHEMBL1860	P10827	3ILZ	91%	99%
Transcription intermediary factor 1-alpha	CHEMBL3108638	O15164	4YBM	91%	96%
HERG	CHEMBL240	Q12809	5VA1	87%	90%
Glycine transporter 2	CHEMBL3060	Q9Y345	N/A	86%	99%
Muscarinic acetylcholine receptor M4	CHEMBL1821	P08173	5DSG	85%	94%
Casein kinase II alpha/beta	CHEMBL3038477	P67870	6TLS	85%	99%
Muscarinic acetylcholine receptor M5	CHEMBL2035	P08912	6OL9	85%	95%
Nuclear receptor ROR-beta	CHEMBL3091268	Q92753	N/A	84,7%	95,5%
Nuclear factor erythroid 2-related factor 2	CHEMBL1075094	Q16236	2FLU	85%	96%
Neuronal acetylcholine receptor; alpha4/beta4	CHEMBL1907591	P30926	6UR8	84%	100%
Kruppel-like factor 5	CHEMBL1293249	Q13887	N/A	83%	86%

Table S3. Molecular docking results of Betti reaction products (**MMZ**-compounds) as well as all the reference ligands.

Drug Target	PDB Code	MMZ-33	MMZ-39	MMZ-45	MMZ-140	MMZ-147	MMZ-167	Reported inhibitor
				**SwissTargetPrediction**				
				Kinases				
**AKT1**	6CCY	-7.03	-7.63	-8.66	-8.05	-6.53	-7.95	-12.46
**AURKB**	4AF3	-7.82	-7.41	-8.36	-8.47	-6.57	-7.25	-9.88
**CDK1**	6GU6	-7.76	-7.50	**-9.02**	-7.93	-7.58	-7.67	-8.41
**CDK2**	2FVD	-6.63	-6.91	**-9.20**	-7.87	-6.37	-7.22	-8.97
**EGFR**	7VRA	-5.71	-6.47	-6.89	-6.65	-5.56	-6.46	-11.34
**FGFR**	5AM6	-5.54	-5.45	-6.58	-6.41	-5.38	-5.68	-7.64
**FLT3**	4RT7	-6.11	-6.29	-7.20	-5.44	-4.73	-5.78	-12.71
**JAK2**	2B7A	-6.75	-7.12	-7.97	-7.41	-6.57	-6.92	-8.60
**JAK3**	3PJC	-7.70	-8.46	-9.35	-8.67	-7.89	-8.54	-9.63
**PLK1**	3FC2	-6.26	-7.09	**-7.68**	-6.57	-5.27	-5.74	-6.32
**PRKCQ**	4Q9Z	-8.21	-7.84	-8.66	-8.33	-8.29	-7.76	-9.50
**RIPK2**	5W5O	-6.58	-6.39	-7.61	-7.07	-6.74	-6.69	-7.72
				Family A/G protein-coupled receptors				
**ADORA1**	6D9H	-7.25	-7.27	**-8.72**	-7.35	-7.49	-7.52	-6.42
				Protease				
**BACE1**	4IVT	-6.72	-7.10	-7.67	-6.93	-6.60	-6.20	-10.40
**BACE2**	2EWY	-7.49	-7.60	-8.63	-8.33	-7.06	-7.68	-10.93
**LTA4H**	5N3W	-6.19	-7.28	-8.10	-6.59	-6.37	-7.12	-8.37
**CAPN1**	2NQG	-5.44	-5.66	**-6.52**	-5.95	-4.94	-5.85	-2.59
**MMP16**	1RM8	-8.74	-8.76	-9.72	-8.13	-7.93	-7.92	-8.60
				Phosphodiesterase				
**PDE2A**	5U7D	-8.45	-8.01	-9.07	-8.37	-7.36	-8.65	-10.30
**PDE5**	2H42	-7.77	-8.44	**-10.31**	-8.00	-8.02	-8.23	-9.62
**PDE4B**	4KP6	-8.30	-8.45	**-10.11**	-8.56	-7.23	-8.11	-8.98
**PDE7A**	4PM0	-7.29	-7.94	**-9.18**	-8.52	-6.83	-7.57	-7.93

**PDE10A**	3WI2	-6.64	-6.71	-8.38	-7.25	-6.96	-6.94	-10.77
				Enzyme				
**HSD17B2**	3HB4	-9.53	-9.96	-12.29	-10.07	-9.22	-9.93	-12.97
**IDH1**	5LGE	-5.96	-5.72	**-6.90**	-6.75	-5.47	-6.14	-6.51
**LDHA**	6MV8	-6.46	-6.05	-6.44	-6.36	-5.30	-5.98	-8.65
				Cytochrome				
**CYP11B1**	6M7X	-7.32	-7.46	-7.94	-6.90	-6.96	-7.33	-6.73
**CYP11B2**	4ZGX	-8.00	-8.51	-9.32	-8.02	-7.76	-7.86	-7.84
				Transcription factor				
**STAT3**	6NUQ	-4.58	-4.93	-5.44	-4.76	-4.13	-4.88	-6.25
				Reader				
**SMARCA2**	5DKH	-6.87	-7.33	-8.05	-5.72	-6.20	-6.64	-8.36
				Transmembrane 1-electron transfer carriers				
**UQCRB**	5NMI	-7.20	-7.54	-8.36	-7.59	-7.08	-7.18	-7.60
				Other cytosolic				
**MCL1**	5FDR	-7.96	-6.75	-8.77	-8.10	-7.05	-7.31	-10.38
				**SuperPred 3.0**				
**APE1**	6BOW	-4.22	-4.34	-4.21	-4.59	-4.10	-3.47	-5.98
**BCHE**	5LKR	-8.78	-8.84	**-9.97**	-8.62	-7.44	-8.45	-7.27
**C5AR**	6C1R	-5.12	-5.62	-7.26	-5.66	-5.34	-5.72	-10.29
**CAPN1**	1ZCM	-5.92	-6.34	**-7.54**	-6.86	-5.69	-6.14	-5.20
**CHRM4**	5DSG	-10.48	-10.71	-10.50	-10.38	-9.55	-9.27	-11.09
**CHRM5**	6OL9	-10.05	-10.22	-10.18	-10.05	-8.83	-9.40	-10.6
**CHRNA4**	6UR8	-5.34	-5.70	-6.75	-6.10	-5.41	-6.17	-5.86
**CK**	6TLS	-7.58	-7.65	**-8.84**	-7.71	-7.21	-7.65	-6.28
**CLK4**	6FYV	-7.57	-7.69	-8.89	-8.12	-7.12	-765	-9.97
**COX2**	5F19	-6.80	-6.76	-7.78	-6.67	-5.94	-6.75	N/D
**CPAR**	6C1R	-5.12	-5.62	-7.26	-5.66	-5.34	-5.72	-10.29
**CPSD**	4OD9	-7.26	-7.64	-8.65	-7.56	-6.66	-7.39	-9.94

**CYP3A4**	5VCC	-5.88	-6.41	-6.87	-6.20	-5.71	-7.07	-21.45
**DUSP3**	3F81	-5.27	-5.55	-6.17	-5.95	-4.61	-5.66	-7.69
**FPR2**	6OMM	-5.95	-5.51	-5.93	-5.51	-4.33	-5.37	-7.20
**GLUT1**	6THA	-6.15	-6.77	**-7.99**	-6.74	-6.44	-6.92	-1.38
**GNAI1**	6N4B	-7.76	-8.31	**-9.77**	-8.28	-7.37	-7.88	-9.25
**HADH2**	2O23	-7.04	-7.96	-8.58	-7.38	-7.59	-6.84	-13.21
**HERG**	5VA1	-3.19	-3.17	-3.54	-3.72	-2.87	-3.11	-5.06
**MDM4**	6Q9Y	-5.81	-5.89	-7.29	-6.13	-5.39	-5.86	-8.32
**NFKB1**	1SVC	-4.73	-4.93	**-5.79**	-5.08	-3.92	-4.89	-2.29
**NR1A1**	3ILZ	-8.55	-7.62	-10.92	-9.97	-8.81	-8.96	-12.91
**NR1I2**	6TFI	-8.82	-8.91	-10.19	-9.15	-8.06	-8.89	-11.21
**PRCP**	3N2Z	-7.25	-6.92	-6.63	-7.48	-4.82	-5.32	-6.51
**PSMA2**	6KWY	-3.38	-3.42	-3.90	-4.30	-3.05	-4.14	-22.93
**SLC1A3**	5LM4	-5.53	-5.49	-6.76	-6.10	-4.65	-5.39	-9.61
**TDP1**	6N0D	-5.82	-5.54	-6.42	-5.61	-4.64	-4.73	-7.67
**TRIM24**	4YBM	-6.14	-6.08	**-7.13**	-6.27	-5.56	-6.57	-5.98
